# Blood–brain barrier penetration prediction enhanced by uncertainty estimation

**DOI:** 10.1186/s13321-022-00619-2

**Published:** 2022-07-07

**Authors:** Xiaochu Tong, Dingyan Wang, Xiaoyu Ding, Xiaoqin Tan, Qun Ren, Geng Chen, Yu Rong, Tingyang Xu, Junzhou Huang, Hualiang Jiang, Mingyue Zheng, Xutong Li

**Affiliations:** 1 Drug Discovery and Design Center, State Key Laboratory of Drug Research, Shanghai Institute of Materia Medica, Chinese Academy of Sciences, 555 Zuchongzhi Road, Shanghai, 201203 China; 2 University of Chinese Academy of Sciences, No. 19A Yuquan Road, Beijing, 100049 China; 3 Nanjing University of Chinese Medicine, 138 Xianlin Road, Nanjing, 210023 China; 4 School of Pharmaceutical Science and Technology, Hangzhou Institute for Advanced Study, UCAS, Hangzhou, 310024 China; 5 Tencent AI Lab, Shenzhen, 518057 China

**Keywords:** Blood–brain barrier penetration, BBBp prediction, Uncertainty estimation

## Abstract

Blood–brain barrier is a pivotal factor to be considered in the process of central nervous system (CNS) drug development, and it is of great significance to rapidly explore the blood–brain barrier permeability (BBBp) of compounds in silico in early drug discovery process. Here, we focus on whether and how uncertainty estimation methods improve in silico BBBp models. We briefly surveyed the current state of in silico BBBp prediction and uncertainty estimation methods of deep learning models, and curated an independent dataset to determine the reliability of the state-of-the-art algorithms. The results exhibit that, despite the comparable performance on BBBp prediction between graph neural networks-based deep learning models and conventional physicochemical-based machine learning models, the GROVER-BBBp model shows greatly improvement when using uncertainty estimations. In particular, the strategy combined Entropy and MC-dropout can increase the accuracy of distinguishing BBB + from BBB − to above 99% by extracting predictions with high confidence level (uncertainty score < 0.1). Case studies on preclinical/clinical drugs for Alzheimer’ s disease and marketed antitumor drugs that verified by literature proved the application value of uncertainty estimation enhanced BBBp prediction model, that may facilitate the drug discovery in the field of CNS diseases and metastatic brain tumors.

**Supplementary Information:**

The online version contains supplementary material available at 10.1186/s13321-022-00619-2.

## Introduction

With the development of society and the aging of the population, central nervous system (CNS) diseases have become the second largest disease after cardiovascular diseases. However, the success rate of clinical candidate CNS drugs is only about 8%, which is quite low compared with the success rate of 20% for cardiovascular diseases [1]. Cancer is also a major disease in the current society, and the success rate of clinical candidates is only about 5% [2]. Worse still, brain metastases are a common route of disease progression in 20% of patients with cancer. The vast majority of patients with brain metastases have a poor prognosis even with the treatment of whole-brain radiation therapy. Second-generation kinase inhibitor with blood–brain barrier (BBB) permeability is believed to be one of the effective treatments of brain metastases [3, 4].

Many potential drugs have been discontinued during their development for clinical use for their insufficient quantity to the CNS because the presence of a BBB. BBB is formed by the endothelial cells of the brain capillaries, which controls the transport of molecules between central nervous system and circulatory system, protects the brain from the damage of toxic compounds and maintains the homeostasis inside the CNS [5]. BBB permeability (BBBp) of compounds is affected by many mechanisms. In the clinical applications of CNS drugs, small lipophilic molecules can cross plasmatic membranes to enter the brain usually by passive diffusion. Some small hydrophilic compounds are recognized by the endogenous influx or efflux transporters, and some large molecules are undergoing transport through endocytic route [6]. Approximately 98% of small molecules cannot cross the BBB [7]. Inappropriate physicochemical properties (PCP) could limit passive diffusion of drugs into the brain [1], and the active efflux transport, especially the ATP binding cassette family (ABC transporters), could decrease the concentration of many drugs in the CNS by pumping them out from brain to blood. Therefore, it puts forward higher requirements for the design of CNS drugs with not only excellent activity, metabolic properties and low toxicity, but also great BBB penetration that makes them reach the CNS with adequate exposure [8].

With increasing experimental data on BBB permeability, attempts have been made to use computational models to predict the BBBp of compounds, which help to minimize the cost of experiments and facilitate high-throughput screening for enormous compounds. In 1980, Levin proposed the best fit model between the BBB permeability coefficient and logP for compounds with molecular weight (MW) less than 400 Da [9]. Since the early 2000s, there have been a large number of in silico BBBp prediction models reported, most of those aimed to find the relationship between scores of molecular physicochemical descriptors and logBB, the unbound brain-to-unbound plasma ratio (K_p,uu_) and BBB ± [10–12]. Most of in silico predictions have been derived from data on the total brain-to-plasma concentration ratio, K_p_, expressed in its logarithmic form i.e. logBB. The logBB value is affected by the extent of plasma protein and brain tissue binding, however, based on “free-drug hypothesis”, K_p,uu_ is more informative [6]. BBB ± is another property used to study the ability of BBBp compounds by dividing compounds into BBB + and BBB − groups based on logBB ratio [13, 14] and CNS activity [15], so that the size of the database can be enlarged.

Although many BBBp models have been reported, the number of compounds used to train is very limited, that always leads to overfitting and misleading on the compounds that is outside the chemical space of training data. Many researchers have focused on minimizing the number of molecular descriptors to avoid overfitting, and meanwhile finding physicochemical properties that are crucial for BBBp of compounds to benefit rational drug design. Zhao et al. used 19 simple molecular descriptors for the analysis of 1593 BBB ± data and showed the importance of hydrogen-bonding properties in modeling BBBp [16]. Gupta et al. built a prediction model “BBB Score”, which consists of stepwise and polynomial piecewise functions. Twenty-two molecular descriptors were studies to describe physicochemical property space, and five descriptors were selected, namely number of aromatic rings, number of heavy atoms, MWHBN (a descriptor related to MW, hydrogen bond acceptor (HBA) and hydrogen bond donor (HBD)), topological polar surface area (TPSA) and pK_a_ [17]. Zhang et al. constructed k-nearest neighbors and support vector machine (SVM) models to predict BBBp using 854 molecular descriptors from different sources, and found that PSA, logP, HBA and HBD are more contributing to the model than others [18]. Yuan et al. showed that the combination of property-based descriptors and molecular fingerprints can significantly improve the performance of SVM-based BBBp prediction model comparing with models using property-based descriptors or molecular fingerprints alone [19]. LightBBB is a BBBp prediction model based on Light Gradient Boosting Machine algorithm with a total of 2432 1D/2D molecular descriptors selected by exclusive feature bundling to avoid overfitting [20]. Alsenan et al. compared kernel PCA, linear PCA, random projection and autoencoder based on BBB dataset with a composed of 6394 property-based descriptors and molecular fingerprints, and proved that dimensionality-reduction techniques can alleviate the overfitting and improve the performance of the model, especially kernel PCA [21]. Roy et al. used 27 molecular descriptors incorporated with 10 molecular solvation energy descriptors based on Kovalenko-Hirata closure (3D-RISM-KH) molecular solvation theory to construct the BBBp prediction model and analyzed the importance of these descriptors by random forest (RF) and gradient boosting machine. A minimum-descriptor-based model with five most important descriptors was obtained, and found it still had good performance [22].

As summarized above, the performance of the above machine learning (ML)-based methods on BBBp prediction depends on the selection of different physicochemical descriptors or molecular fingerprints and subsequent feature extraction, which requires prior knowledge and always prone to bias when selecting features manually. Deep learning (DL) techniques can automatically select optimal features from the provided dataset. In particular, graph neural networks (GNNs) try to learn molecular representation directly from molecular graphs to perform property prediction tasks. Most of these newly proposed GNNs have shown excellent performance based on the evaluation on a benchmark BBBp dataset from MoleculeNet [23]. Xiong et al. proposed Attentive FP for molecular representation by introducing a graph attention mechanism. Attentive FP enabled the graph neural network to extract not only atomic local information but also nonlocal interactions at the intramolecular level to learn additional interactions which affect the overall properties of molecules [24]. Wang et al. built a multichannel gated recurrent unit architecture to extract molecular features both at the node level and molecule level, so as to cover more elaborate molecular information [25], which may be beneficial to the task of molecular property prediction. Hu et al. established a pre-trained model for unsupervised learning on the graph representation of molecules before using the model to predict molecular properties, which can learn from more unlabeled molecular structures, and to a certain extent, improve the performance of downstream prediction tasks [26]. Recently, Rong et al. proposed a molecular representation framework GROVER [27] based on transformer framework. The strategy of randomly selecting the number of hops can be adapted to different types of datasets, thereby further expanding the learnable molecular datasets. Moreover, node/edge-level and graph-level self-supervised tasks were constructed to learn rich structural and semantic information from a large number of unlabeled molecules in pre-training process. The well-trained GROVER model was fine-tuned with molecules with task-specific labels and used for BBBp prediction as a graph-level task.

For those complex GNNs, simply focusing on the improvement of metrics on the existing benchmark datasets may lead to the neglect of their applicability in practical applications. The deviations between the modeling dataset and the real-world observations will bring the uncertainty for the predictions. For example, in MoleculeNet [23], the molecules defined as BBB + are about three times as much as the BBB − , while it is estimated that 98% of molecules cannot pass through BBB in the real chemical space [7]. Generally speaking, by adding more high-quality experimental data, the applicability of models can be improved. However, it is not easy to increase the size of BBBp datasets, because measuring the value of BBBp is often complicated, time-consuming and costly. In this circumstance, introducing uncertainty estimation [28] can expand the application domain of the prediction models [29–31]. There are some general approaches for both ML- and DL-based models. For example, Shannon entropy, as a measure of information, also allows us to make accurate statements and perform calculations about the confidence of models’ prediction truth [32]. Multiple initialization (Multi-initial) means initializing the model parameters many times randomly to get several independent models trained with same dataset and thus the variances of the prediction results are considered as the uncertainties of the prediction [33]. Recently, there have been some custom-made algorithms of uncertainty estimation to suit the framework of DL model. Monte Carlo dropout (MC-dropout) has been proposed as an approximation of Bayesian neural networks (BNNs) [34, 35] in deep neural networks [36, 37] to reduce computational consumption, which only need to apply dropout in existing model during inference to get the distribution of prediction results. Besides, compared with the model-agnostic measurement of the distance of molecular representation in a feature space, like fingerprints, the latent space is a more intuitive way to estimate the uncertainty, which does not require retraining the model. Recently, Janet et al. proposed the distance in latent space as a new uncertainty estimation method, which specifically calculates Euclidean distance of each test molecule to the nearest training set molecule in the final layer latent space of deep neural network [38].

In this study, we first analyze the existing BBBp benchmark dataset in MoleculeNet [23], and collect the additional molecules as an external benchmark dataset to evaluate the performance of different types of BBBp prediction models. Furthermore, due to overfitting of DL models and the deviation of training data distribution from the real-world distribution, we introduce uncertainty estimation to quantitatively evaluate the reliability of prediction results and determine the optimal combination of different uncertainty estimation methods. We examine our strategy on preclinical/clinical drugs for Alzheimer’s disease and marketed antitumor drugs, and verify it by literature. Ideally, selecting the molecules with certainty for the further wet experiments will reduce unnecessary costs and thus benefit real-world application of in silico BBBp prediction model.

## Results and discussion

### Benchmark dataset analysis and new benchmark dataset collection

**Fig. 1:**
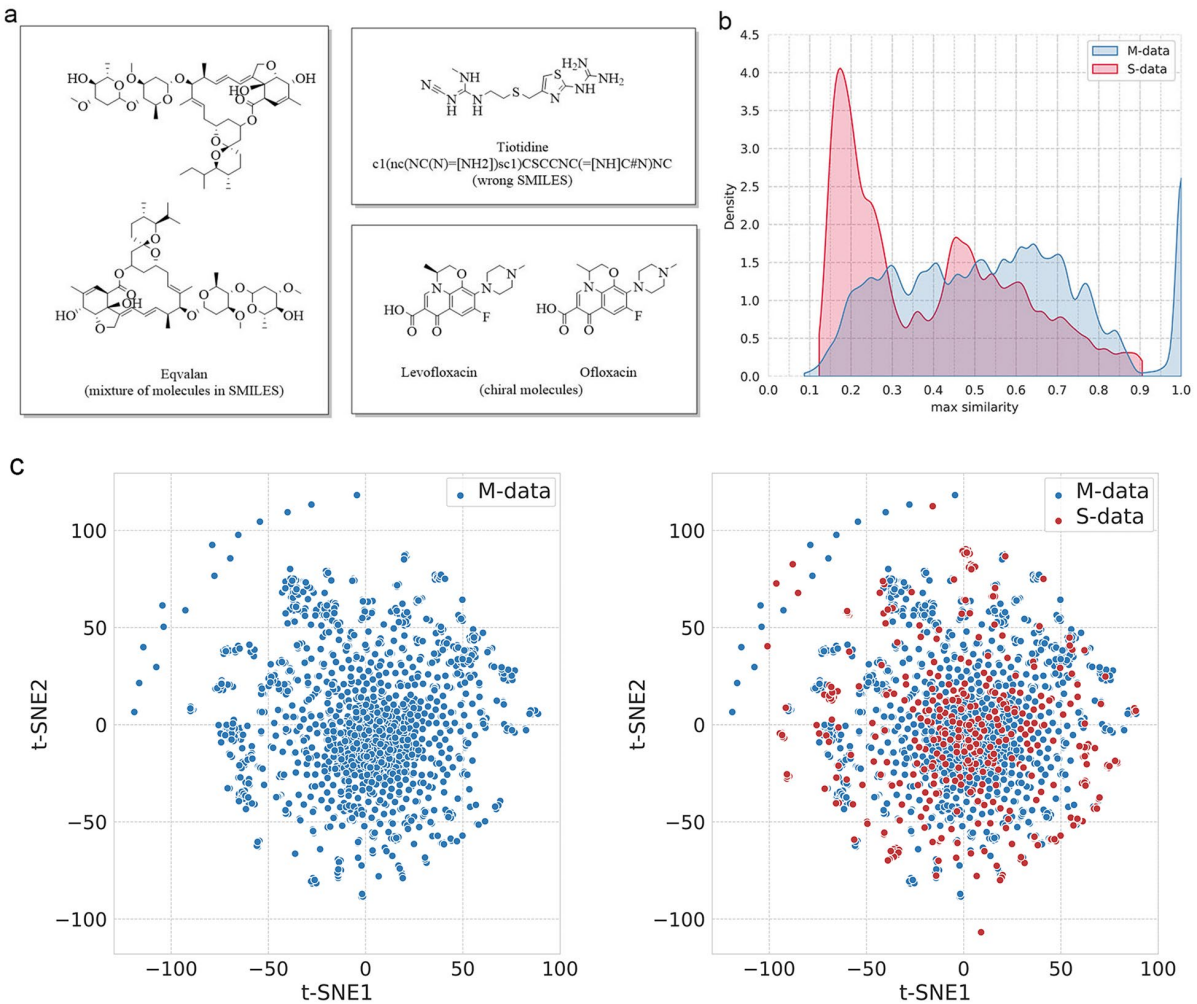
Analyzing molecules’ defects in M-data and the distribution of chemical space of S-data and M-data. **a** A list of defective molecules in M-data. **b** The distribution of max similarities inside M-data (blue) and max similarity of each molecule in S-data relative to M-data based on ECFP4 (red). **c** t-SNE distribution of M-data and S-data based on ECFP4

MoleculeNet [23] is a benchmark for molecular machine learning that curates multiple public datasets focus on different levels of properties of molecules, including a BBBp dataset [39]. The BBBp dataset contains 2053 molecules that were collected from previous works discussing BBB penetration [14, 16, 18, 40]. The molecules are defined as BBB + or BBB − according to logBB ≥  − 1 or logBB <  − 1 (K_p_ ≥ 0.1 or K_p_ < 0.1). Although MoleculeNet-BBBp dataset is a relative standard and comprehensive collection of BBBp data, some defects should be pointed out (Fig. 1a). (1) It contains a number of mixtures of molecules like eqvalan. (2) It contains molecules with wrong SMILES that can’ t be identified by RDKit, like tiotidine. (3) It contains duplicate molecules. For example, ofloxacin is exactly the same molecule named 40,730, and it can also be a duplicate of levofloxacin as some models cannot recognize chiral molecules. Even introducing the information of chirality, enantiomers in the dataset like ofloxacin/levofloxacin can lead to inflated model performance. Thus, we have further processed MoleculeNet-BBBp dataset by removing salts and solvents, neutralizing, and extracting the single molecule with the largest molecular weight from SMILES. After standardization and removal of duplication, we have an updated BBBp benchmark, called M-data, that contains 1937 molecules, comprising 1476 BBB + and 461 BBB − for further analysis.

After that, we curated an independent supplementary dataset to test the existing BBBp prediction models, named S-data. On the one hand, we supplemented hundreds of CNS and non-CNS drugs that not included in M-data based on Anatomical Therapeutic Chemical (ATC) classification system [15, 41]. ATC annotation is a reasonable inference about whether drugs can cross the human BBB, and can be used to test the generalization performance of computational BBBp model that build on the heterogeneous experiment data of other species. On the other hand, we collected previous reported compounds from literature [42–44] that are not contained in M-data, and newly released compounds from ChEMBLdb25 [45] that have measured logBB or K_p_. Finally, the new benchmark dataset S-data contains 527 molecules that are assigned to 395 BBB + and 132 BBB − according to logBB ≥  − 1/logBB <  − 1 or CNS/non-CNS drugs. To analyzing the distribution of chemical space of S-data and M-data, we use the Tanimoto similarities and t-distributed stochastic neighbor embedding (t-SNE) based on molecular fingerprint ECFP4 [46]. Figure 1b shows the distribution of max internal similarities in M-data and the distribution of max similarity of each molecule in S-data relative to M-data. The majority of molecules in S-data are structurally different from M-data with low max similarities range from 0.1 to 0.3. Meanwhile, the visual analysis of t-SNE shows that some molecules in S-data do not overlap with M-data (Fig. 1c). Therefore, S-data can be used as an independent benchmark dataset to measure the generalization ability of BBBp prediction models build on M-data for the deviation in chemical space.

### Validation of BBBp prediction models on independent S-data

We evaluate the performance of existing in silico BBBp prediction models, including BBB score, RF, multi-layer perceptron (MLP), and two the state-of-the-art (SOTA) GNN algorithms, namely Attentive FP and GROVER. First of all, to verify we have correctly rebuilt the GNN-based models, we implement threefold cross validations on these models using M-data as it has done in GROVER. As a self-supervised GNNs model that pre-trained with a large number of drug-like molecules, GROVER shows the best performance with excited metric scores that are in accord with it was reported in its research (the area under ROC curve (ROC_AUC) = 0.976, the area under ROC curve (PRC_AUC) = 0.994, matthews correlation coefficient (MCC) = 0.842 and balanced accuracy (BACC) = 0.910). Close behind GROVER is Attentive FP, and both of the GNN-based model significantly outperforms RF and MLP (Additional file 1: Table S1).

**Fig. 2:**
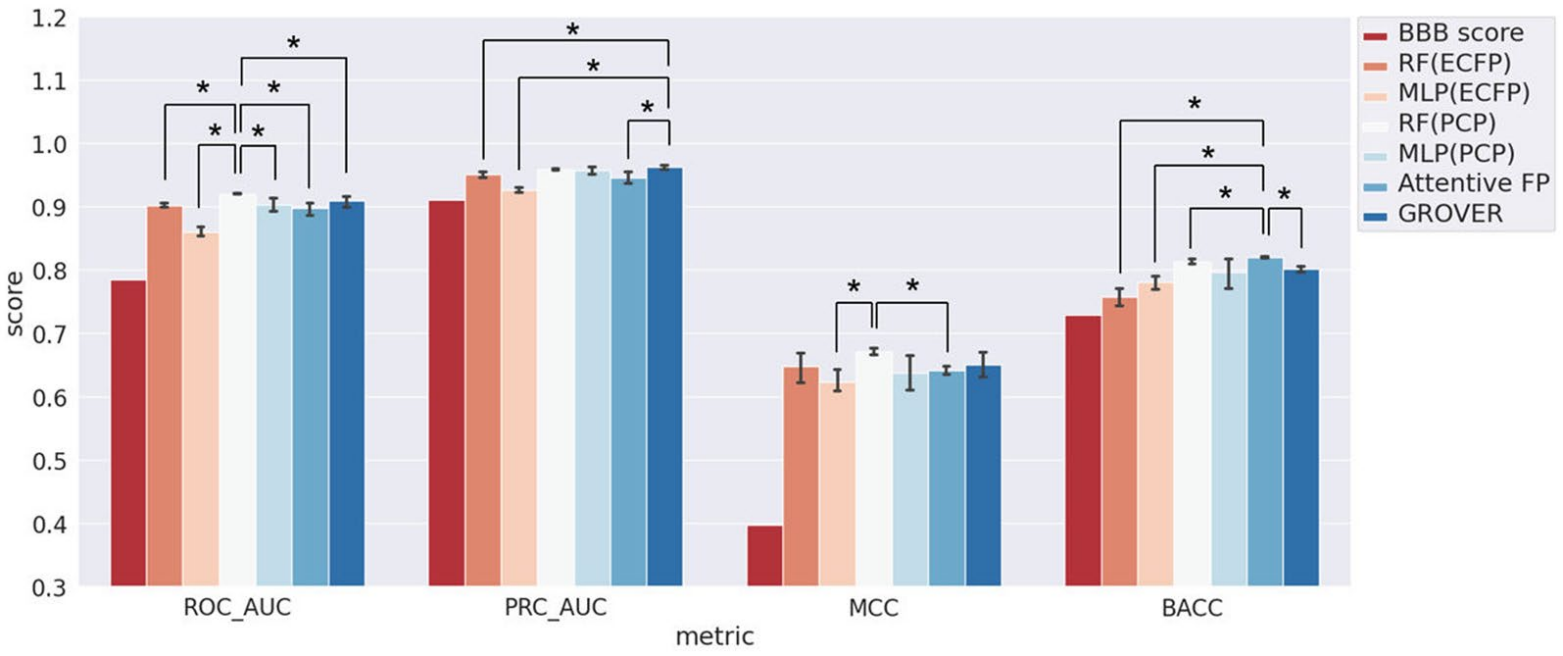
Prediction performance on S-data by BBBp prediction models. Each histogram with an error bar indicates the mean and variance of 5 runs of the model, respectively. Statistical t-tests were applied between the model with the highest metric score and others, and statistically significant test results were noted (*p < 0.05)

Next, we focus on the evaluation on S-data. Except BBB score that was a series of predefined linear functions, models were trained by M-data with 5 times runs of different initialization and evaluated by S-data. The implementation details are described in Method, and the performance is shown in Fig. 2 and Additional file 1: Table S2. Above all, compared to the evaluation results on M-data, the slump in all metric scores of these models substantiates the independence of S-data. Inevitably, BBB score shows much worse generalization ability on the independent S-data, as its selection of molecular descriptors relies on prior knowledge. Among models, GROVER shows highest PRC_AUC score than others, and significantly higher than RF(ECFP) and MLP(ECFP) model. Attentive FP shows best performance and significantly exceed RF(PCP) when measured by BACC, but RF(PCP) also could be the best according to ROC_AUC and MCC. Actually, except ECFP-based ML models, RF(PCP), MLP(PCP), Attentive FP and GROVER show moderate and comparable performance on independent testing dataset, i.e. S-data. This rises a suspicion that the exciting metric scores on M-data of these models, especially the SOTA GNN-based models, are only attributed to overfitting.

In fact, it is expected that GNN-based models could learn more task-specific features directly from topological structure of molecules. Thus, we implement a test based on substrates of transporters. Using physicochemical properties as molecular features to predict drug’s BBBp is based on an assumption that the majority of drugs could get across the BBB by passive diffusion [47], but active transport mechanisms also exercise considerable influence over the drug concentration in the CNS. The substrates of transporters [48–50] in S-data were extracted to evaluate the performance of these models (Additional file 1: Tables S3 and S4). As a self-supervised-based model that has been pre-trained with a tremendous number of drug-like molecules, we expected that GROVER could distinguish substrates of transporters better than others. However, as the confusion matrix for the prediction results that shown in Table 1, the right predictions (true positive (TP) and true negative (TN)) of PCP-based ML models are on a par with GROVER, as well as Attentive FP. Molecular graph features or pre-training could not give GNN-based models distinct advantages on the prediction of substrates of transporters.

**Table 1:** Confusion matrix of model predictions on 27 substrates in S-data

	RF(PCP)	MLP(PCP)	Attentive FP	GROVER
TP	10	11	11	10
FN	3	2	2	3
FP	1	2	3	1
TN	13	12	11	13

Overall, the moderate improvements of GNN-based models are inconsistent with the hype in their publications. The insufficient modeling data may lead to the inability to give full play to the advantages of DL models in the scenario of BBBp prediction.

### Commonly used uncertainty estimation in BBBp prediction

In view of the above-mentioned results on independent S-data, the performance of in silico BBBp prediction models is not only limited by algorithms but also by data. Overfitting may undermine the generalization performance of the SOTA DL models with complicated architecture and high-capacity. Furthermore, the deviation between modeling data and real-world data could affect the model’s practicability. In fact, it is estimated that about 98% of small molecules are BBB − impenetrable [7], whereas in MoleculeNet-BBBp dataset, BBB + molecules are over three times as much as BBB − molecules. The practicability of an in silico model that too closely fit to a dataset like that, despite the high accuracy, seems to be rather dubious. In DL models, uncertainty estimation is increasingly important component of assessing prediction truth for its potential to secure its practicability, and would be the high road to circumvent the data obstacle. Here, aiming at bring DL-based BBBp models into play, we focus on how uncertainty impacts the performance of learning from insufficient BBBp data, in particular under the framework of GNNs.

We implemented five proposed algorithms of uncertainty estimation in GROVER-BBBp and Attentive FP-BBBp models. And we also explored uncertainty on PCP-based RF and MLP rather than ML models based on ECFP as the former showed much better performance on BBBp dataset than the latter. Entropy, MC-dropout and Multi-initial can capture the prediction uncertainty of a classification model without any change to its architecture; FPsDist and LatentDist measuring the distance of molecular representation in a feature space and the latent space respectively, are more intuitive ways to estimate the uncertainty as they don’t involve re-running or re-training the model. Besides, random method was applied as a baseline for the comparison of different uncertainty estimation methods (See “Methods” Section for more details).

**Fig. 3:**
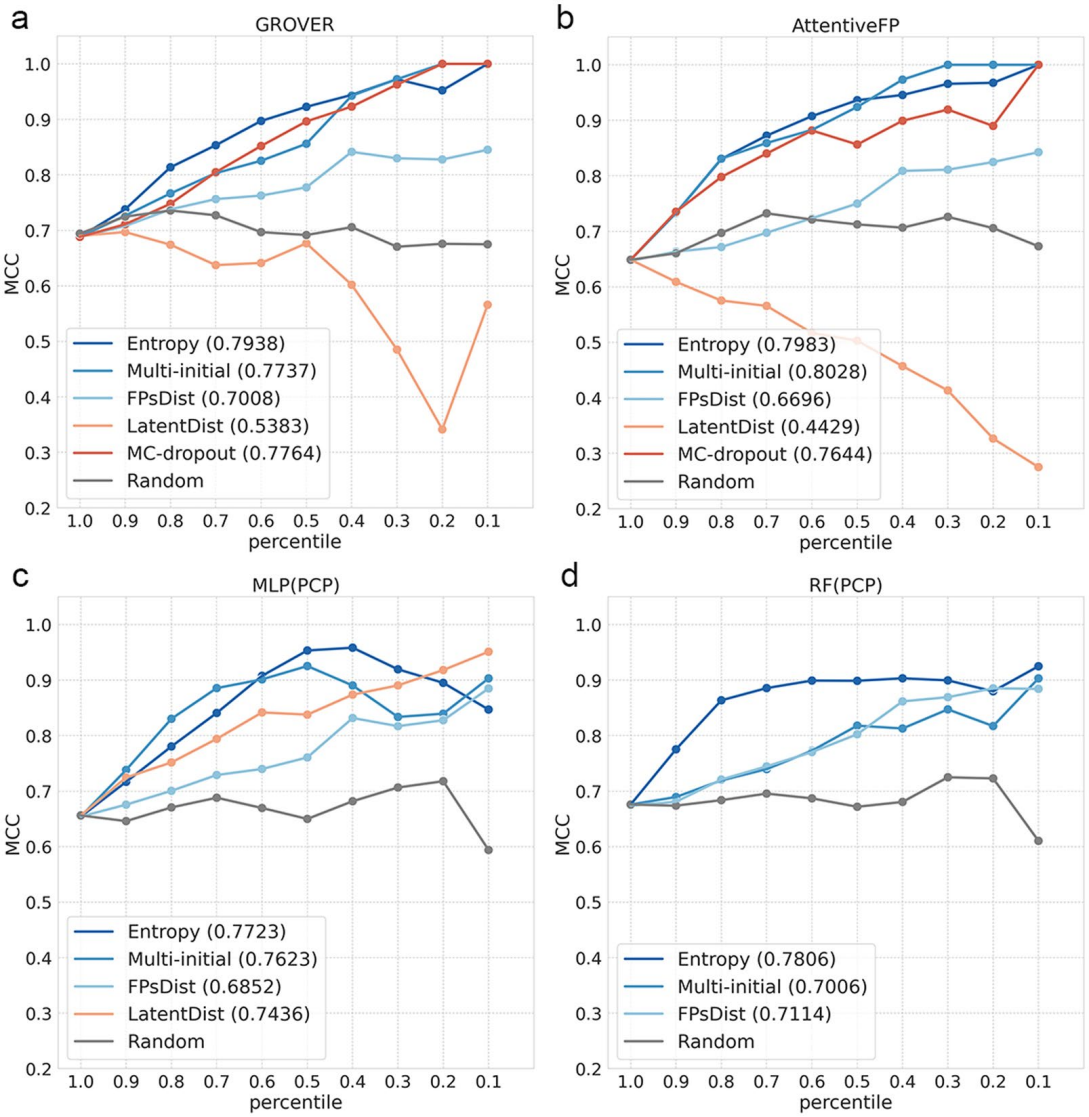
Prediction performance by introducing different uncertainty estimation methods for BBBp prediction models. **a** The MCC curves for different uncertainty estimation methods in GROVER, namely Entropy, MC-dropout, Multi-initial, FPsDist, LatentDist and random method. The x-axis is the proportion of remaining compounds in S-data when the compounds with high uncertainty are sequentially discarded, and y-axis is corresponding MCC of the BBBp prediction model. The MCC_AUC is shown in parentheses. **b** The MCC curves for different uncertainty estimation methods in Attentive FP. **c** The MCC curves for different uncertainty estimation methods in MLP(PCP). **d** The MCC curves for different uncertainty estimation methods in RF(PCP)

In order to explore the correlation between uncertainty level and prediction correctness, we discarded predictions with top 10% uncertainty in S-data sequentially and calculated the MCC of the remaining (Fig. 3), since MCC is a more stringent metric for imbalance dataset like BBBp. First of all, in all models, the flat trend of the random method indicating that the uncertainty values assigned randomly cannot lead to model improvement, and thus can served as baseline. Entropy, MC-dropout and Multi-initial show relatively better performance with the higher under curve area of MCC (MCC_AUC) than distance-based methods i.e. FPsDist and LatentDist. In particular, for GNN-basesd model i.e. GROVER-BBBp and Attentive FP-BBBp, Entropy, MC-dropout and Multi-initial lead to relatively steady improvements in MCC with decreases in quantity of high-uncertainty compounds, whereas the MCC curves for MLP(PCP) and RF(PCP) cannot keep rising with these methods. By comparison, FPsDist can supplement relatively robust fingerprint information, and thus shows moderate upward swings in all of models. The trends of LatentDist on GNN-based models are the opposite of that on MLP(PCP), probably because of the more serious overfitting to M-data of GNNs than MLP. The closer distance of latent embedding between the training and testing data could exacerbate overfitting and thus cause misleading. Overall, compared to PCP-based ML models, introducing uncertainty estimation to GNN-BBBp models improves performance more greatly, in which the prediction performance measured by MCC can reach 1 for the remaining 10% of the most certain molecules. Furthermore, we used a variety of drug-like datasets to demonstrate the reliability of uncertainty-enhanced GROVER model, and found that Entropy, MC-dropout and Multi-initial can enhance the prediction performance of GROVER robustly in applied 9 binarized datasets from admetSAR [51] (Additional file 1: Tables S8–S19 and Fig. S1).

**Fig. 4:**
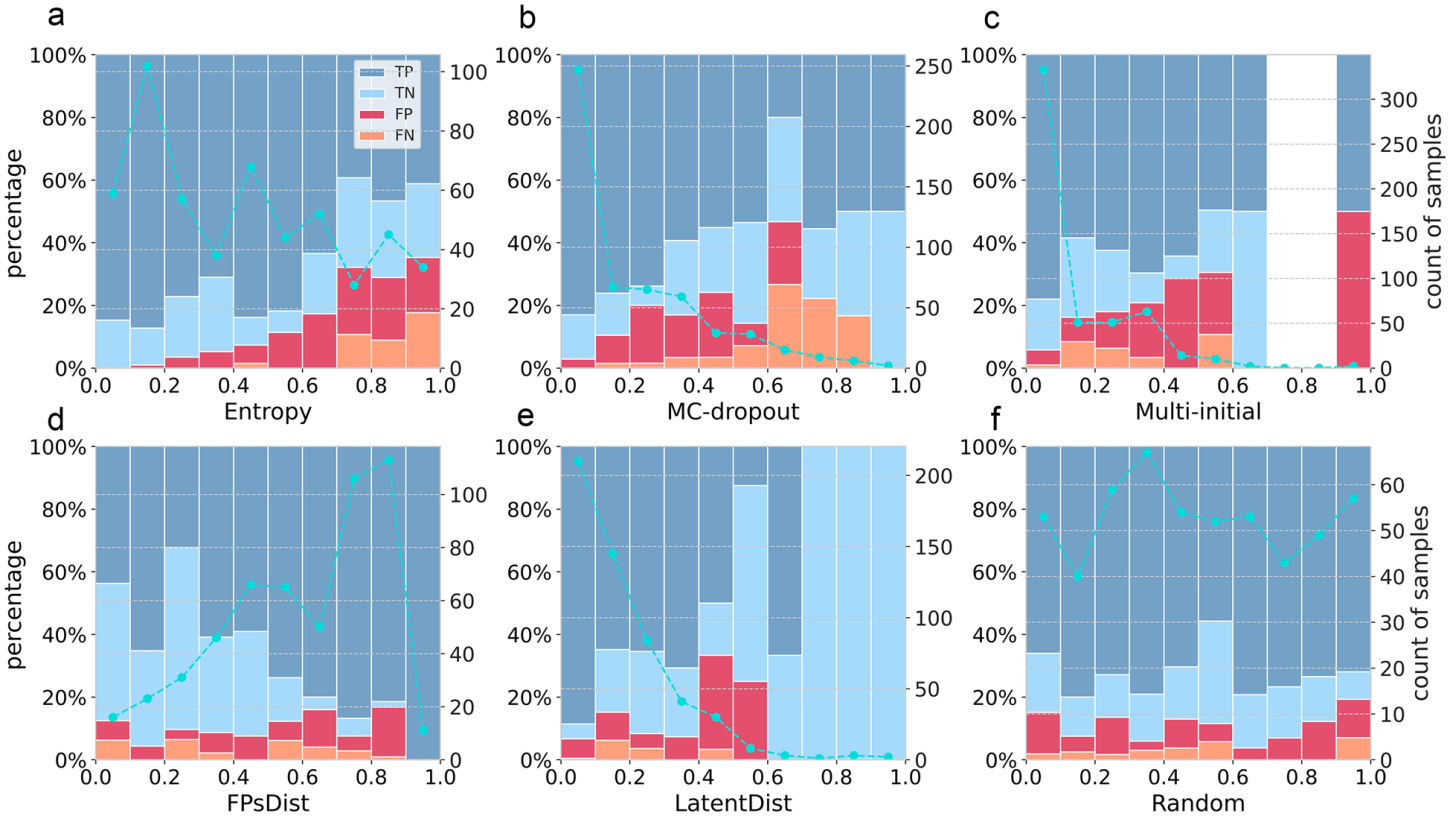
Prediction results from GROVER-BBBp model on S-data within different uncertainty ranges, and corresponding numbers of molecules. **a** Entropy method. **b** MC-dropout method. **c** Multi-initial method. **d** FPsDist method. **e** LatentDist method. **f** Random method

Considering that GROVER-BBBp model showed relatively good performance when enhanced by varied uncertainty estimation methods containing DL-specific LatentDist and MC-dropout, we focus on GROVER to analyze how the different uncertainty estimation methods and their combinations effect the performance of BBBp prediction task. Figure 4 shows the percentages of molecules in S-data with the prediction of TP, TN, false positive (FP) and false negative (FN) within different uncertainty ranges, and also their total numbers. As shown in Fig. 4a, entropy values of these molecules are comparatively well-distributed in each interval, and as entropy increasing, the proportion of molecules that are incorrectly predicted (FP and FN) gradually increases. When comes to MC-dropout and Multi-initial (Fig. 4b and c), though they tend to give low uncertainty to most of the predicted molecules that makes the distributions of them are non-uniform and even discontinued, the upward trends of the proportion of misprediction can be also seen when uncertainty get higher. Whereas, the clear trend is not shown in FPsDist and LatentDist, and the latter is even inferior to random method. These observations are in accord with the growth curves of the performance of GROVER that enhanced by these uncertainty estimation methods in Fig. 3a.

### Optimal combination strategy of uncertainty estimation in BBBp prediction

Next, we attempt to explore whether the ensemble of these uncertainty estimation methods would provide more steadily enhancement in GROVER-BBBp model. The combined uncertainty values were calculated by moderated-Z (modZ) weighted average algorithm, and the corresponding MCC_AUC values are summarized in Additional file 1: Table S5 and partly shown in Table 2. It is found that the combination of Entropy and MC-dropout obtains a highest MCC_AUC among all combination scheme, and outperforms using Entropy method alone, which is the single method of highest MCC_AUC for GROVER. But MCC_AUC cannot keep climbing when add Multi-initial, FPsDist or LatentDist further (Table 2).

**Table 2:** Model performance of various combinations of uncertainty estimation methods in GROVER-BBBp model

Entropy	MC-dropout	Multi-initial	FPsDist	LatentDist	MCC_AUC
√					0.7938
	√				0.7764
		√			0.7737
			√		0.7008
				√	0.5383
√	√				**0.7965**
√	√	√			0.7879
√	√		√		0.7956
√	√			√	0.7771

The highest value is highlighted in bold

**Fig. 5:**
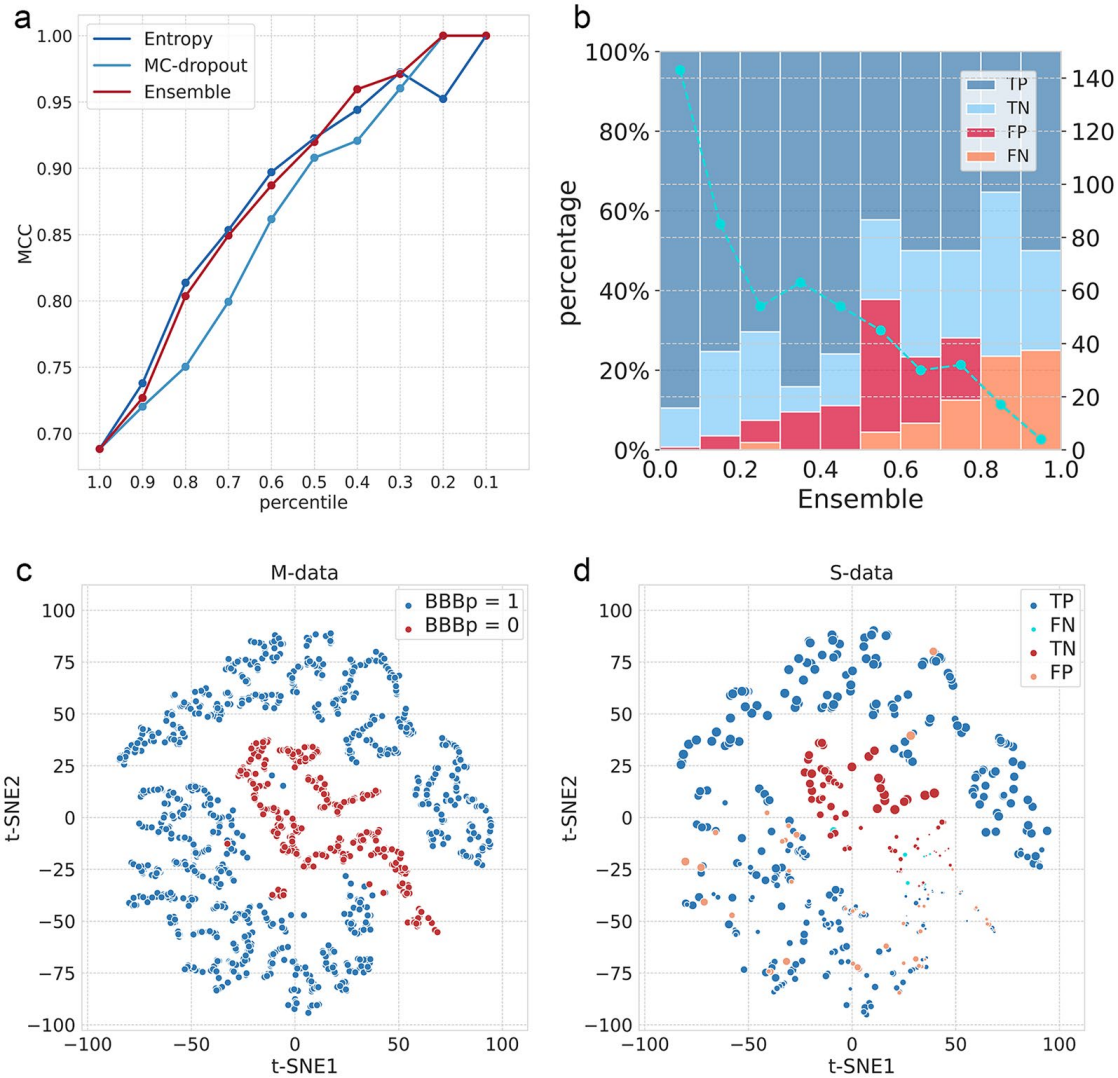
Prediction performance by introducing ensemble uncertainty and t-SNE distribution for molecules in M-data and S-data. **a** The MCC curves of Entropy, MC-dropout and ensemble of them. **b** Prediction results of molecules in S-data within different range of the ensemble uncertainty, and corresponding numbers of molecules. **c** t-SNE distribution of M-data based on latent representation of GROVER. **d** t-SNE distribution of S-data based on latent representation of GROVER, and the size of the point represents the uncertainty of the prediction. The larger the size of the point, the smaller the uncertainty value

Figure 5a shows the MCC curves of Entropy method, MC-dropout method and the ensemble of them. We conclude that the ensemble of Entropy and MC-dropout is a robust strategy for enhancing the model prediction, that has also been verified on other 9 drug-like datasets (Additional file 1: Table S8–S19 and Fig. S1). In particular, when remaining 20% of molecules with lower uncertainty, using the ensemble method shows better performance than using Entropy alone, as the latter shows a slightly decrease here. This improvement is necessary for the reason that molecules with the most certain prediction have priorities over others for further experimental verification. Figure 5b shows the prediction results under the different range of the ensemble uncertainty. For ensemble uncertainty below 0.5 (left side), only 5.4% molecules (22 of 408 molecules) are wrongly predicted. Remarkably, the prediction accuracy is above 99% when ensemble uncertainty values are less than 0.1, as there is only one FP prediction in 144 molecules in range 0–0.1. Therefore, the uncertainty value is a reliable guide for the usage of the GROVER-BBBp model. Predicted BBB + molecules with lower uncertainty values can be put into experimental verification with greater confidence but molecules with uncertainty values above 0.5 should be treated with circumspection.

In addition, t-SNE is used to visualize how uncertainty estimation works in GROVER-BBBp model. Figure 5c, d show the t-SNE plots of latent representation of GROVER for molecules in M-data and S-data, respectively. By introducing uncertainty estimation in S-data prediction, the molecules with high uncertainty are mainly concentrated at the junction of BBB + and BBB − . Thus, uncertainty estimation can help GROVER provide more reliable and practical decisions to further distinguish inseparable molecules in the hidden space.

### Application of BBBp prediction model enhanced by uncertainty estimation

To verify the practicability of GROVER-BBBp model enhanced by uncertainty estimation, we first predicted preclinical/clinical drugs for Alzheimer’s disease, and the results have been summarized in Table 3. FPS-ZM1, a specific RAGE inhibitor to block Aβ binding to the V domain of RAGE, could readily cross the BBB and considered as a candidate drug for the treatment of Alzheimer’s disease [52, 53]. Consistently, FPS-ZM1 is predicted to be able to cross the BBB in our model, and the uncertainty value is less than 0.5. Tarenflurbil is predicted to be able to cross the BBB with uncertainty of 0.6328, but its Phase III clinical trial failed due to its poor brain delivery efficiency [54]. In fact, in situ rat brain perfusion experiments have shown that tarenflurbil can rapidly cross the BBB in the absence of plasma protein, but plasma protein binding significantly limits the free plasma fraction of tarenflurbil, and further leads to decrease in the concentration into the brain [55, 56]. As the model was built based on logBB, what only considered the total concentration rather than unbound concentration of drugs in brain and plasma, the high plasma protein binding rate may be the reason for the wrong prediction of tarenflurbil. On the other hand, although mis-predicted, tarenflurbil shows an uncertainty value greater than 0.5, which indicates the low reliability of the prediction result and avoids our excessive trust in it.

**Table 3:** A list of prediction results with uncertainty of clinical drugs and marketed antitumor drugs

Drug	Structure*	Predicted probability	Uncertainty	Potential indications
FPS-ZM1		0.9761	0.1964	Alzheimer’s disease
Tarenflurbil		0.7881	0.6328	Alzheimer’s disease
Niraparib		0.9563	0.3676	Carcinoma ovarian, fallopian tube cancer and peritoneal carcinoma
Alectinib		0.9484	0.3907	Non-small cell lung cancer (NSCLC) metastatic, ALK-positive
Encorafenib		0.2529	0.7017	Melanoma with BRAF mutation, colorectal cancer with BRAF V600 mutation
Osimertinib		0.4864	0.7486	NSCLC advanced, metastatic, EGFR mutation

*Structures of drugs used in model are stripped of chirality

Moreover, despite the advances in the treatment of many cancers, CNS tumors and brain metastases still pose significant challenges, partially because few of the antitumor drugs can penetrate the BBB to reach specific targets in brain. As a test of GROVER-BBBp model, we performed BBBp prediction for some small molecule inhibitors (SMIs) that have been marketed for tumor treatment, and verified by literature. The prediction results of some molecules with available records related to BBBp are shown in Table 3 (the full list of antitumor SMIs and corresponding predictions in Additional file 1: Table S6). In general, because these antitumor SMIs are structurally different from training molecules, their uncertainty values are higher than those in S-data, which are all greater than 0.3. Among these drugs, niraparib and alectinib are predicted as BBB + with high confidence with uncertainty of 0.3676 and 0.3907, respectively. Niraparib is an FDA-approved poly (ADP-ribose) polymerase-1/-2 inhibitor for anticancer treatment [57, 58]. Niraparib (probability of 0.9563 with uncertainty of 0.3676) has shown its ability in a BRCA2-mutant intracranial tumor model [59], and its brain to plasma exposure ratio is about 0.3, that corroborates the confident BBB + prediction [57]. Alectinib (probability of 0.9484 with uncertainty of 0.3907) has been approved by FDA to treating ALK-positive NSCLC patients for both systemic and intracranial disease [60–62]. Preclinical studies have also proved that alectinib has a high brain-to-plasma ratio and drug permeability in vitro [63]. Encorafenib (probability of 0.2529 with uncertainty of 0.7017) is an example of TN infer. It is a selective BRAF inhibitor which has been approved for treating melanoma, with a brain-to-plasma ratio of approximately 0.004 in mice model [64, 65].

The GROVER-BBBp model enhanced by uncertainty estimation can correctly predict whether molecules can cross the BBB for most clinical or marketed drugs, and further quantitatively indicate the reliability of the model’s prediction results. However, it still has some limitations. Although logBB is a widely used parameter to quantify the brain permeability of drugs, the K_p,uu_ is a more ideally endpoint than K_p_, as it removes the interference of plasma protein and brain tissue binding that affect K_p_. However, publicly available K_p,uu_ data is not enough for de novo building of in silico BBBp model. Therefore, we have used Friden’s K_p,uu_ dataset [44] to fine-tune GROVER-BBBp model to predict K_p,uu_, expecting to fix the model’s bias toward K_p_ to some extent. Dataset collected from Colclough [3] and Kim’s [66] works was constructed to test the fine-tuned model externally, in which molecules defined as BBB + or BBB − according to K_p,uu_ ≥ 0.1 or K_p,uu_ < 0.1. The results show that the fine-tuned model can correct some misprediction (Additional file 1: Table S7). For example, osimertinib is EGFR inhibitor for the treatment of advanced NSCLC patients with EGFR-mutated and demonstrated efficacy against stable or asymptomatic CNS metastases [67]. Recent experiment has shown that K_p,uu_ of osimertinib in-vivo rat is 0.21 [3] that characterizes its permeability [68]. In the original prediction results, GROVER-BBBp model incorrectly predicted it as BBB − (probability of 0.4864 with uncertainty of 0.7486), while the latest fine-tuned model successfully predicted it as BBB + , with the corresponding prediction probability of 0.6952. We can expect future exploration on K_p,uu_ may provide a more comprehensive prospect for the prediction of BBBp.

## Conclusions

In this study, a newly collected independent dataset S-data was used as a benchmark dataset to evaluate the performance of BBBp prediction models, which contained a total of 527 molecules with 395 BBB + and 132 BBB − respectively. Among various BBBp models, conventional PCP-based ML models and GNN-based models exhibit moderate and comparable prediction performance, which suggests that overfitting to a bias training dataset could undermine the generalization ability of the SOTA GNN-based models with complicated architecture. Thus, in order to secure the practicability of in silico BBBp prediction models, we introduced uncertainty estimation to quantify the reliability of prediction results of these models. GNN-BBBp models enhanced by uncertainty estimation show greater improvement than PCP-based ML models, and we find that using the combination of Entropy and MC-dropout for uncertainty estimation in GROVER-BBBp model is the optimal strategy. Based on this strategy, the BBBp potential of many drugs of Alzheimer’s disease and cancer were successfully predicted with a quantitative estimation of prediction reliability and verified by literature.

In conclusion, our study makes the first attempt to offer insights into prediction uncertainty into ML- and DL-based BBBp prediction model. Uncertainty estimation helps determine how much we can trust a prediction result, enhanced by that, the proposed BBBp in silico model can speed up the high-throughput screening and lead optimization of BBBp molecules, and beneficial to the discovery of drugs for the treatment of CNS diseases and malignant tumors with brain metastasis.

## Methods

### Data sets

The BBBp dataset collected from MoleculeNet [23] contains a total of 2053 BBB + /BBB − molecules defining based on whether logBB ≥  − 1 (K_p_ ≥ 0.1). After removing salts and solvents, neutralizing, and extracting the single molecule with the largest molecular weight from SMILES, the preprocessed and deduplicated M-data contained 1937 molecules, including 1476 BBB + and 461 BBB − . The new benchmark dataset was derived from CNS and non-CNS drugs based on ATC [15, 41], and compounds with measured logBB in previous literature [42–44] or ChEMBLdb25 [45], and preprocessed in the same way as M-data to get S-data with 527 molecules (395 BBB + and 132 BBB −). The K_p,uu_ dataset from Friden [44] was divided into BBB + /BBB − according to whether K_p,uu_ ≥ 0.1, comprising 24 BBB + and 17 BBB − . And external test set from Colclough [3] and Kim’s [66] works contained 18 molecules (4 BBB + and 14 BBB −).

### Models for BBBp prediction

In this study, we used different types of BBBp prediction models, including BBB Score [17], conventional ML models RF and MLP, and GNN-based models Attentive FP [24] and GROVER [27].

For BBB score, physicochemical descriptors like MW, HBA, HBD, TPSA and number of aromatic rings were calculated by RDKit toolkit and pK_a_ was obtained with Epik module of Maestro. The final BBB score was summed according to the functions associated with the above descriptors, and molecules were considered as BBB + when the scores ≥ 4, otherwise labeled as BBB − .

ML models often use physical- or chemical-property descriptors or molecular fingerprints as the inputs. In this study, we used simple networks RF and MLP, and molecular fingerprint ECFP4 or physicochemical properties used in GROVER were calculated as the representation input to the ML models.

For RF models, three hyperparameters were considered including ‘n_estimators’, ‘max_depth’, and ‘criterion’. The best group of these hyperparameters for RF(ECFP) is set ‘n_estimators’ to 100, ‘max_depth’ to 50, and ‘criterion’ to ‘entropy’. The best group of these hyperparameters for RF(PCP) is set ‘n_estimators’ to 250, ‘max_depth’ to 20, and ‘criterion’ to ‘gini’.

For MLP models, four hyperparameters ‘hidden_layer_sizes’, ‘max_iter’, ‘batch_size’ and ‘learning_rate_init’ were considered. In MLP(ECFP) model, the best group of these hyperparameters is set ‘hidden_layer_sizes’ to [1000, 500], ‘max_iter’ to 3000, ‘batch_size’ to 64 and ‘learning_rate_init’ to 0.0001. In MLP(PCP) model, the best group of these hyperparameters is set ‘hidden_layer_sizes’ to [1500, 1000, 500], ‘max_iter’ to 1000, ‘batch_size’ to 16 and ‘learning_rate_init’ to 0.0001.

For retraining Attentive FP model, we followed the optimal parameters given in article [24]. And on the basic of pre-trained GROVER model [27], we further fine-tuned the downstream BBBp prediction model on M-data following the determined hyper-parameters from the article.

### Uncertainty estimation methods

In this study, we implemented five uncertainty estimation methods, including Entropy, MC-dropout, Multi-initial, FPsDist and LatentDist. At the same time, the random method was used as the benchmark to compare different uncertainty estimation methods.

The random method is achieved by randomly assigning value between 0 and 1 to each molecule in S-data as an uncertainty value.

Schwill proposed that entropy can be used as a measure of uncertainty [32]. And it is the most classical way to measure the uncertainty of classification models. Specifically, the definition of Shannon entropy used in this study is as follows:

1 $$\mu =-\sum_{c}{{p}_{c} logp}_{c}$$

where $\mu$ is entropy measure, and ${p}_{c}$ corresponds to the probability value of each class in model’s output, which is multiplied by corresponding logarithmic value and finally summed. In BBBp prediction model, we used the probability value of the model’s output to get the entropy value, that is, the uncertainty, and the larger this value, the greater the uncertainty of the model.

MC-dropout in deep neural networks has been proved to be used as an approximation of BNN [36, 37]. In practice, there is no need to change the framework of existing DL models, but only need to apply dropout during inference. Finally, the variances of different prediction results obtained by multiple inferences are taken as the values of uncertainty estimation.

Multi-initial is a basic method for uncertainty estimation. The model is trained several times independently with different initialization, and the variances of its results can also be considered as uncertainties of the prediction.

For FPsDist, we calculated the Tanimoto distance of the molecules in S-data relative to the nearest-neighbor molecule in M-data using ECFP4 [69]. LatentDist is a new uncertainty estimation method by measuring the distance in latent space, which specifically calculates Euclidean distance of each test molecule to the nearest training set molecule in the final layer latent space of deep neural network [38].

For the combined uncertainty values obtained by different uncertainty estimation methods, modZ weighted average algorithm is used. Specifically, considering entropy uncertainty as a standard value, Spearman’s rank correlation coefficients between other uncertainty values and entropy values are used as weights of uncertainty obtained by other uncertainty estimation methods, and uncertainty values with different weights are averaged to obtain the final combined uncertainty values.

### Evaluation metrics

For assessment of BBBp prediction model performance, several metrics are evaluated. The ROC curve takes false positive rate as the x-axis and true positive rate (recall) as the y-axis, and the area under ROC curve is called ROC_AUC for short in this study. Similarly, the PRC curve uses recall as the x-axis and precision as the y-axis, and the area under PRC curve is abbreviated as PRC_AUC in this study. The larger ROC_AUC value and PRC_AUC value, the better the performance of the BBBp prediction model. The false positive rate, recall, and precision are defined as follows:

2 $$false\, positive\, rate=\frac{FP}{TN+FP}$$

3 $$recall/sensitivity=\frac{TP}{TP+FN}$$

4 $$precision=\frac{TP}{TP+FP}$$

Specifically, BBBp dataset has the problem of data imbalance, BACC and MCC are used in this study to evaluate classification model performance, which are relatively balanced metrics considering TP, TN, FP and FN simultaneously. These two metrics are defined as:

5 $$BACC=(\frac{TP}{TP+FN}+\frac{TN}{TN+FP})/2$$

6 $$MCC=\frac{TP\times TN-FP\times FN}{\sqrt{(TP+FP)(TP+FN)(TN+FP)(TN+FN)}}$$

## Supplementary Information

**Additional file 1: Table S1** Model performance on threefold cross validations using M-data. **Table S2** Model performance on S-data trained by M-data with 5 times runs. **Table S3** Substrates in S-data. **Table S4** Model performance on substrates in S-data. **Table S5** Model performance of all combinations of uncertainty estimation methods in GROVER-BBBp model. **Table S6** A list of antitumor SMIs and corresponding prediction results in GROVER-BBBp model. **Table S7** Prediction results for external dataset collected from Colclough and Kim’s works in GROVER-BBBp model. **Table S8** The details of 9 drug-like datasets from admetSAR. **Table S9** Model performance on fivefold cross-validations of 9 drug-like datasets in GROVER model. **Table S10** Model performance on test set of 9 drug-like datasets in GROVER model. **Fig. S1** Prediction performance by introducing different uncertainty estimation methods in 9 GROVER models. **Table S11** Model performance of various combination of uncertainty estimation methods on fathead minnow toxicity dataset in GROVER model. **Table S12** Model performance of various combination of uncertainty estimation methods on tetrahymena pyriformis toxicity dataset in GROVER model. **Table S13** Model performance of various combination of uncertainty estimation methods on AMES mutagenicity dataset in GROVER model. **Table S14** Model performance of various combination of uncertainty estimation methods on hERG inhibitor (II) dataset in GROVER model. **Table S15** Model performance of various combination of uncertainty estimation methods on CYP3A4 substrates dataset in GROVER model. **Table S16** Model performance of various combination of uncertainty estimation methods on CYP1A2 inhibitor dataset in GROVER model. **Table S17** Model performance of various combination of uncertainty estimation methods on Caco-2 permeability dataset in GROVER model. **Table S18** Model performance of various combination of uncertainty estimation methods on P-gp inhibitor (I) dataset in GROVER model. **Table S19** Model performance of various combination of uncertainty estimation methods on P-gp inhibitor (II) dataset in GROVER model.

## Data Availability

All data and code to build the models are provided at:https://github.com/tongxiaochu/BBB_uncertainty_project.

## Abbreviations

ALK: Anaplastic lymphoma kinase
ATC: Anatomical Therapeutic Chemical
BACC: Balanced accuracy
BBB: Blood–brain barrier
BBBp: Blood–brain barrier permeability
BNNs: Bayesian neural networks
CNS: Central nervous system
DL: Deep learning
EGFR: Epidermal growth factor receptor
FN: False negative
FP: False positive
GNNs: Graph neural networks
HBA: Hydrogen bond acceptor
HBD: Hydrogen bond donor
MCC: Matthews correlation coefficient
ML: Machine learning
MLP: Multi-layer perceptron
MW: Molecular weight
NSCLC: Non-small cell lung cancer
RAGE: Receptor of advanced glycation end products
ROTB: Rotatable bond
RF: Random forest
SMIs: Small molecule inhibitors
SVM: Support vector machine
TN: True negative
TP: True positive
TPSA: Topological polar surface area
t-SNE: T-distributed stochastic neighbor embedding
