# Supplementary material for: Blood–brain barrier penetration prediction enhanced by uncertainty estimation
Source: J Cheminform. 2022 Jul 7;14:44. doi: 10.1186/s13321-022-00619-2 (PMC9264551; doi:10.1186/s13321-022-00619-2)
Supplement: Supplementary file 1 — Additional file 1: Table S1 Model performance on threefold cross validations using M-data. Table S2 Model performance on S-data trained by M-data with 5 times runs. Table S3 Substrates in S-data. Table S4 Model performance on substrates in S-data. Table S5 Model performance of all combinations of uncertainty estimation methods in GROVER-BBBp model. Table S6 A list of antitumor SMIs and corresponding prediction results in GROVER-BBBp model. Table S7 Prediction results for external dataset collected from Colclough and Kim’s works in GROVER-BBBp model. Table S8 The details of 9 drug-like datasets from admetSAR. Table S9 Model performance on fivefold cross-validations of 9 drug-like datasets in GROVER model. Table S10 Model performance on test set of 9 drug-like datasets in GROVER model. Fig. S1 Prediction performance by introducing different uncertainty estimation methods in 9 GROVER models. Table S11 Model performance of various combination of uncertainty estimation methods on fathead minnow toxicity dataset in GROVER model. Table S12 Model performance of various combination of uncertainty estimation methods on tetrahymena pyriformis toxicity dataset in GROVER model. Table S13 Model performance of various combination of uncertainty estimation methods on AMES mutagenicity dataset in GROVER model. Table S14 Model performance of various combination of uncertainty estimation methods on hERG inhibitor (II) dataset in GROVER model. Table S15 Model performance of various combination of uncertainty estimation methods on CYP3A4 substrates dataset in GROVER model. Table S16 Model performance of various combination of uncertainty estimation methods on CYP1A2 inhibitor dataset in GROVER model. Table S17 Model performance of various combination of uncertainty estimation methods on Caco-2 permeability dataset in GROVER model. Table S18 Model performance of various combination of uncertainty estimation methods on P-gp inhibitor (I) dataset in GROVER model. Table S19 Model performanc [file 13321_2022_619_MOESM1_ESM.docx]

Additional file

Blood–Brain Barrier Penetration Prediction Enhanced by Uncertainty Estimation

*Xiaochu Tong^1,2^**,* *Dingyan Wang^1,2^, Xiaoyu Ding^1,2^, Xiaoqin Tan^1,2^, Qun Ren^3,1^,* *Geng Chen^,1,2,4^, Yu Rong^5^, Tingyang Xu^5^, Junzhou Huang^5^, Hualiang Jiang^1,2^, Mingyue Zheng^1,2,*^ and Xutong Li^1,2,*^*

^1^Drug Discovery and Design Center, State Key Laboratory of Drug Research, Shanghai Institute of Materia Medica, Chinese Academy of Sciences, 555 Zuchongzhi Road, Shanghai 201203, China

^2^University of Chinese Academy of Sciences, No. 19A Yuquan Road, Beijing 100049, China

^3^Nanjing University of Chinese Medicine, 138 Xianlin Road, Nanjing 210023, China

^4^School of Pharmaceutical Science and Technology, Hangzhou Institute for Advanced Study, UCAS, Hangzhou 310024, China

^5^Tencent AI Lab, Shenzhen, 518057, China

**Table of Contents:**

| **Table S1** Model performance on threefold cross validations using M-data. | S3 |
| --- | --- |
| **Table S2** Model performance on S-data trained by M-data with 5 times runs. | S3 |
| **Table S3** Substrates in S-data. | S3 |
| **Table S4** Model performance on substrates in S-data. | S5 |
| **Table S5** Model performance of all combinations of uncertainty estimation methods in GROVER-BBBp model. | S5 |
| **Table S6** A list of antitumor SMIs and corresponding prediction results in GROVER-BBBp model. | S5 |
| **Table S7** Prediction results for external dataset collected from Colclough and Kim’s works in GROVER-BBBp model. | S10 |
| **Table S8** The details of 9 drug-like datasets from admetSAR. | S11 |
| **Table S9** Model performance on fivefold cross-validations of 9 drug-like datasets in GROVER model. | S11 |
| **Table S10** Model performance on test set of 9 drug-like datasets in GROVER model. | S12 |
| **Fig. S1** Prediction performance by introducing different uncertainty estimation methods in 9 GROVER models. | S13 |
| **Table S11** Model performance of various combination of uncertainty estimation methods on fathead minnow toxicity dataset in GROVER model. | S14 |
| **Table S12** Model performance of various combination of uncertainty estimation methods on tetrahymena pyriformis toxicity dataset in GROVER model. | S14 |
| **Table S13** Model performance of various combination of uncertainty estimation methods on AMES mutagenicity dataset in GROVER model. | S14 |
| **Table S14** Model performance of various combination of uncertainty estimation methods on hERG inhibitor (II) dataset in GROVER model. | S15 |
| **Table S15** Model performance of various combination of uncertainty estimation methods on CYP3A4 substrates dataset in GROVER model. | S15 |
| **Table S16** Model performance of various combination of uncertainty estimation methods on CYP1A2 inhibitor dataset in GROVER model. | S15 |
| **Table S17** Model performance of various combination of uncertainty estimation methods on Caco-2 permeability dataset in GROVER model. | S16 |
| **Table S18** Model performance of various combination of uncertainty estimation methods on P-gp inhibitor (I) dataset in GROVER model. | S16 |
| **Table S19** Model performance of various combination of uncertainty estimation methods on P-gp inhibitor (II) dataset in GROVER model. | S16 |
| **References** | S17 |

**Table S1** Model performance on threefold cross validations using M-data.

|  | **ROC_AUC** | **PRC_AUC** | **MCC** | **BACC** |
| --- | --- | --- | --- | --- |
| RF(ECFP) | 0.9250 ± 0.0170 | 0.9670 ± 0.0078 | 0.6910 ± 0.0380 | 0.7940 ± 0.0270 |
| MLP(ECFP) | 0.9070 ± 0.0071 | 0.9590 ± 0.0043 | 0.6600 ± 0.0520 | 0.7920 ± 0.0340 |
| RF(PCP) | 0.9410 ± 0.0120 | 0.9770 ± 0.0064 | 0.7190 ± 0.0380 | 0.8300 ± 0.0220 |
| MLP(PCP) | 0.9200 ± 0.0037 | 0.9680 ± 0.0029 | 0.6840 ± 0.0160 | 0.8250 ± 0.0150 |
| Attentive FP | 0.9660 ± 0.0150 | 0.9870 ± 0.0063 | 0.7920 ± 0.0640 | 0.8780 ± 0.0300 |
| GROVER | **0.9760 ± 0.0090** | **0.9940 ± 0.0020** | **0.8420 ± 0.0270** | **0.9100 ± 0.0320** |

**Table S2** Model performance on S-data trained by M-data with 5 times runs.

|  | **ROC_AUC** | **PRC_AUC** | **MCC** | **BACC** |
| --- | --- | --- | --- | --- |
| BBB score | 0.7844 | 0.9111 | 0.3975 | 0.7293 |
| RF(ECFP) | 0.902 ± 0.0036 | 0.951 ± 0.0053 | 0.6479 ± 0.0266 | 0.7575 ± 0.0158 |
| MLP(ECFP) | 0.86 ± 0.0082 | 0.9258 ± 0.0047 | 0.6243 ± 0.02 | 0.7811 ± 0.0123 |
| RF(PCP) | **0.9199 ± 0.0007** | 0.959 ± 0.0015 | **0.6717 ± 0.0065** | 0.8137 ± 0.0045 |
| MLP(PCP) | 0.9024 ± 0.0119 | 0.9569 ± 0.0066 | 0.6381 ± 0.0317 | 0.7969 ± 0.0264 |
| Attentive FP | 0.8971 ± 0.0108 | 0.9463 ± 0.0105 | 0.6414 ± 0.0074 | **0.8197 ± 0.0015** |
| GROVER | 0.9088 ± 0.0095 | **0.9628 ± 0.0039** | 0.6508 ± 0.0221 | 0.8012 ± 0.0053 |

**Table S3** Substrates [1-3] in S-data.

| **SMILES** | **BBB+/-** | **Name** |
| --- | --- | --- |
| CC(C)CC(N)C(=O)NC(CCCN)C(=O)NC(C(=O)NC(CCCN)C(=O)N1CCCC1C(=O)NC(C=O)Cc1ccccc1)C(C)C | 0 | gramicidin-j2 |
| CCC1NC(=O)C2CCCN2C(=O)C(Cc2ccccc2)NC(=O)C(CC(C)C)NC(=O)C(CCCN)NC(=O)C(C(C)C)NC(=O)C2CCCN2C(=O)C(Cc2ccccc2)NC(=O)C(CC(C)C)NC(=O)C(CCCN)NC1=O | 0 | gramicidin-s-2 |
| CCC(NC(=O)C1CCCN1C(=O)C(Cc1ccccc1)NC(=O)C(N)CC(C)C)C(=O)NC(CCCN)C(=O)NC(CC(C)C)C(=O)NC(Cc1ccccc1)C(=O)N1CCCC1C(=O)NC(CC)C(=O)NC(C=O)CCCN | 0 | gramicidin-s-3 |
| CN(C)C1C(O)=C(C(N)=O)C(=O)C2(O)C(O)=C3C(=O)c4c(O)cccc4C(C)(O)C3CC12 | 0 | tetracycline |
| COC1CC(OC2C(C)OC(OC3C(C)=CCC4CC(CC5(CCC(C)C(C(C)C)O5)O4)OC(=O)C4C=C(C)C(O)C5OCC(=CC=CC3C)C45O)CC2OC)OC(C)C1O | 0 | eqvalan (ivermectin-ii) |

**Table S3** Substrates [1-3] in S-data. (continued)

| **SMILES** | **BBB+/-** | **Name** |
| --- | --- | --- |
| CNC(=NC#N)NCCSCc1nc[nH]c1C | 0 | cimetidine |
| Cc1cc(NC(=O)C2=C(O)c3ccccc3S(=O)(=O)N2C)no1 | 0 | isoxicam |
| CN1CCC23c4c5ccc(O)c4OC2C(OC2OC(C(=O)O)C(O)C(O)C2O)C=CC3C1C5 | 0 | morphine |
| O=c1c(-c2ccc(O)cc2)coc2cc(O)ccc12 | 1 | daidzein |
| CC(C)CN(CC(O)C(Cc1ccccc1)NC(=O)OC1CCOC1)S(=O)(=O)c1ccc(N)cc1 | 1 | amprenavir-d4 |
| CN1CCC23c4c5ccc(OC6OC(C(=O)O)C(O)C(O)C6O)c4OC2C(O)C=CC3C1C5 | 0 | M3G |
| COC1C(OC(N)=O)C(O)C(Oc2ccc3c(O)c(NC(=O)c4ccc(O)c(CC=C(C)C)c4)c(=O)oc3c2C)OC1(C)C | 0 | novobiocin |
| NS(=O)(=O)c1cc2c(cc1Cl)N=CNS2(=O)=O | 0 | chlorothiazide |
| CC(C)(c1ccc(O)cc1)c1ccc(O)cc1 | 1 | bisphenol A |
| COc1ccc2[nH]c(S(=O)Cc3ncc(C)c(OC)c3C)nc2c1 | 0 | omeprazole |
| CN1C(C(=O)Nc2ccccn2)=C(O)c2sccc2S1(=O)=O | 0 | tenoxicam |
| CC(C)(C)NCC(O)COc1ccccc1C#N | 1 | bunitrolol |
| COc1ccc(CCNCCCC(C#N)(c2ccc(OC)c(OC)c2)C(C)C)cc1OC | 1 | norverapamil |
| COC(=O)C1C(=O)C=C(Nc2cccc([N+](=O)[O-])c2)CC1C | 1 | DM-44 |
| Cc1nc2n(c(=O)c1CCN1CCC(c3noc4cc(F)ccc34)CC1)CC(O)CC2 | 1 | 7-hydroxy risperidone |
| N=C(N)NC(=O)c1nc(Cl)c(N)nc1N | 0 | amiloride |
| O=C(NCCN1CCN(c2cccc3c2OCC(CO)O3)CC1)c1ccc(F)cc1 | 1 | flesinoxan |
| COC(=O)C1C(=O)C=C(Nc2ccc(Cl)cc2)CC1C | 1 | add 196022 |
| Nc1nc2c(ncn2COC(CO)CO)c(=O)[nH]1 | 1 | ganciclovir |
| COc1ccc(OC(F)(F)F)cc1CNC1CCCNC1c1ccccc1 | 1 | 145742-28-5 |
| O=C1CN(N=Cc2ccc([N+](=O)[O-])o2)C(=O)N1 | 1 | nitrofurantoin |
| Cc1cc2c(s1)Nc1ccccc1N=C2N1CCN(C)CC1 | 1 | olanzapine |

**Table S4** Model performance on substrates in S-data.

|  | **ROC_AUC** | **PRC_AUC** | **MCC** | **BACC** |
| --- | --- | --- | --- | --- |
| RF(PCP) | **0.9725** | **0.9660** | **0.7096** | 0.8489 |
| MLP(PCP) | 0.8956 | 0.8668 | 0.7033 | **0.8516** |
| Attentive FP | 0.9396 | 0.9393 | 0.6319 | 0.8159 |
| GROVER | 0.8956 | 0.8967 | **0.7096** | 0.8489 |

**Table S5** Model performance of all combinations of uncertainty estimation methods in GROVER-BBBp model.

| **Entropy** | **MC-dropout** | **Multi-initial** | **FPsDist** | **LatentDist** | **MCC_AUC** |
| --- | --- | --- | --- | --- | --- |
| √ | √ |  |  |  | **0.7965** |
| √ | √ |  | √ |  | 0.7956 |
| √ | √ | √ | √ |  | 0.7943 |
| √ |  | √ | √ |  | 0.7938 |
| √ |  |  |  |  | 0.7938 |
| √ |  | √ |  |  | 0.7934 |
| √ | √ | √ |  |  | 0.7879 |
|  | √ | √ | √ |  | 0.7873 |
| √ | √ | √ | √ | √ | 0.7866 |
| √ | √ | √ |  | √ | 0.7847 |
| √ |  | √ | √ | √ | 0.7830 |
| √ | √ |  | √ | √ | 0.7823 |
|  | √ | √ |  |  | 0.7805 |
| √ |  | √ |  | √ | 0.7804 |
| √ | √ |  |  | √ | 0.7771 |
|  | √ |  |  |  | 0.7765 |
|  |  | √ |  |  | 0.7737 |
| √ |  |  | √ |  | 0.7722 |
|  | √ | √ | √ | √ | 0.7712 |
|  | √ | √ |  | √ | 0.7632 |
|  | √ |  | √ |  | 0.7599 |
|  |  | √ | √ |  | 0.7583 |
|  |  | √ | √ | √ | 0.7491 |
| √ |  |  | √ | √ | 0.7482 |
|  |  |  | √ |  | 0.7008 |
|  |  |  | √ | √ | 0.6944 |
|  | √ |  | √ | √ | 0.6775 |
|  |  | √ |  | √ | 0.6348 |
| √ |  |  |  | √ | 0.5742 |
|  | √ |  |  | √ | 0.5483 |
|  |  |  |  | √ | 0.5383 |

**Table S6** A list of antitumor SMIs and corresponding prediction results in GROVER-BBBp model.

| **SMILES** | **Drug** | **Predicted probability** | **Uncertainty** |
| --- | --- | --- | --- |
| CNCc1ccc(-c2[nH]c3cc(F)cc4c3c2CCNC4=O)cc1 | Rucaparib | 0.9510 | 0.3608 |
| NC(=O)c1cccc2cn(-c3ccc(C4CCCNC4)cc3)nc12 | Niraparib | 0.9563 | 0.3676 |
| CCc1cc2c(cc1N1CCC(N3CCOCC3)CC1)C(C)(C)c1[nH]c3cc(C#N)ccc3c1C2=O | Alectinib | 0.9484 | 0.3907 |
| C=CC(=O)N1CCCC(n2nc(-c3ccc(Oc4ccccc4)cc3)c3c(N)ncnc32)C1 | Ibrutinib | 0.9019 | 0.5306 |
| N#CCC(C1CCCC1)n1cc(-c2ncnc3[nH]ccc23)cn1 | Ruxolitinib | 0.8574 | 0.5553 |
| CCOc1cc2ncc(C#N)c(Nc3ccc(Cc4ccccn4)c(Cl)c3)c2cc1NC(O)C=CCN(C)C | Neratinib | 0.1103 | 0.5717 |
| CCN1C(=O)N(c2c(F)c(OC)cc(OC)c2F)Cc2cnc3[nH]c(CN4CCOCC4)cc3c21 | Pemigatinib | 0.8914 | 0.5812 |
| Cc1cnc(Nc2ccc(OCCN3CCCC3)cc2)nc1Nc1cccc(S(=O)(=O)NC(C)(C)C)c1 | Fedratinib | 0.1215 | 0.5942 |
| CN1CCC(NC(=O)Nc2ccc(C#N)cc2)CC1c1nc2ccccc2[nH]1 | Glasdegib | 0.8666 | 0.5992 |
| CS(=O)(=O)c1ccc(C(=O)Nc2ccc(Cl)c(-c3ccccn3)c2)c(Cl)c1 | Vismodegib | 0.8559 | 0.6040 |
| CNC(=O)c1ccccc1Sc1ccc2c(C=Cc3ccccn3)n[nH]c2c1 | Axitinib | 0.8441 | 0.6132 |
| C=CC(=O)N1CCC(C2CCNc3c(C(N)=O)c(-c4ccc(Oc5ccccc5)cc4)nn32)CC1 | Zanubrutinib | 0.8826 | 0.6198 |
| CCN(CC)CCNC(=O)c1c(C)[nH]c(C=C2C(=O)Nc3ccc(F)cc32)c1C | Sunitinib | 0.8611 | 0.6217 |
| CS(=O)(=O)CCNCc1ccc(-c2ccc3ncnc(Nc4ccc(OCc5cccc(F)c5)c(Cl)c4)c3c2)o1 | Lapatini | 0.1512 | 0.6234 |
| Cc1cc(Nc2ncc(Cl)c(Nc3ccccc3S(=O)(=O)C(C)C)n2)c(OC(C)C)cc1C1CCNCC1 | Ceritinib | 0.2035 | 0.6396 |
| COc1cc2c(Nc3ccc(Br)cc3F)ncnc2cc1OCC1CCN(C)CC1 | Vandetanib | 0.8119 | 0.6500 |
| Cc1cc(Nc2ncnc3ccc(NC4=NC(C)(C)CO4)cc23)ccc1Oc1ccn2ncnc2c1 | Tucatinib | 0.1927 | 0.6540 |
| CC1CCCC(=CCC(OC(=O)CC(C(C(=O)C(C1O)C)(C)C)O)C(=CC2=CSC(=N2)C)C)C | Epothilone D | 0.1566 | 0.6552 |
| Cc1nc(NC(=O)N2CCCC2C(N)=O)sc1-c1ccnc(C(C)(C)C(F)(F)F)c1 | Alpelisib | 0.8645 | 0.6579 |
| CC1Oc2cc(cnc2N)-c2c(nn(C)c2C#N)CN(C)C(=O)c2ccc(F)cc21 | Lorlatinib | 0.8232 | 0.6594 |

**Table S6** A list of antitumor SMIs and corresponding prediction results in GROVER-BBBp model. (continued)

| **SMILES** | **Drug** | **Predicted probability** | **Uncertainty** |
| --- | --- | --- | --- |
| Cc1ccc(C(=O)Nc2ccc(CN3CCN(C)CC3)c(C(F)(F)F)c2)cc1C#Cc1cnc2cccnn12 | Ponatinib | 0.7922 | 0.6757 |
| COc1c(OCCCN2CCOCC2)ccc2c1N=C(NC(=O)c1cnc(N)nc1)N1CCN=C21 | Copanlisib | 0.8227 | 0.6842 |
| CNC(=O)c1ccc(-c2cnc3ncc(Cc4ccc5ncccc5c4)n3n2)cc1F | Capmatinib | 0.7456 | 0.6870 |
| Cn1cc(-c2cc3c(N4CCN(c5ncc(C(C)(N)c6ccc(F)cc6)cn5)CC4)ncnn3c2)cn1 | Avapritinib | 0.7621 | 0.6886 |
| O=C(c1cc(Cc2n[nH]c(=O)c3ccccc23)ccc1F)N1CCN(C(=O)C2CC2)CC1 | Olaparib | 0.7829 | 0.6906 |
| CC(=O)Nc1cccc(-n2c(=O)n(C3CC3)c(=O)c3c(Nc4ccc(I)cc4F)n(C)c(=O)c(C)c32)c1 | Trametinib | 0.5462 | 0.6956 |
| CN1CCN(c2ccc(Nc3ncc4cc5n(c4n3)C3(CCCCC3)CNC5=O)nc2)CC1 | Trilaciclib | 0.7324 | 0.6985 |
| COC(=O)NC(C)CNc1nccc(-c2cn(C(C)C)nc2-c2cc(Cl)cc(NS(C)(=O)=O)c2F)n1 | Encorafenib | 0.2529 | 0.7017 |
| COC1(C(=O)NC(C)c2ccc(-n3cc(F)cn3)nc2)CCC(c2nc(C)cc(Nc3cc(C)[nH]n3)n2)CC1 | Pralsetinib | 0.3971 | 0.7020 |
| COc1cc2nccc(Oc3ccc(NC(=O)Nc4cc(C)on4)c(Cl)c3)c2cc1OC | Tivozanib | 0.4188 | 0.7021 |
| Cc1ccc(Nc2nccc(N(C)c3ccc4c(C)n(C)nc4c3)n2)cc1S(N)(=O)=O | Pazopanib | 0.3589 | 0.7030 |
| Cn1ncnc1C1c2n[nH]c(=O)c3cc(F)cc(c23)NC1c1ccc(F)cc1 | Talazoparib | 0.6246 | 0.7064 |
| CC(Oc1cc(-c2cnn(C3CCNCC3)c2)cnc1N)c1c(Cl)ccc(F)c1Cl | Crizotinib | 0.7285 | 0.7076 |
| CCC(Nc1ncnc2[nH]cnc12)c1nc2cccc(F)c2c(=O)n1-c1ccccc1 | Idelalisib | 0.5173 | 0.7073 |
| CC(C)Oc1ccc(-c2nn(C(C)c3oc4ccc(F)cc4c(=O)c3-c3cccc(F)c3)c3ncnc(N)c23)cc1F | Umbralisib | 0.5709 | 0.7095 |
| COc1cc2nccc(Oc3ccc(NC(=O)C4(C(=O)Nc5ccc(F)cc5)CC4)cc3)c2cc1OC | Cabozantinib | 0.6893 | 0.7108 |
| CC(C)(C)c1nc(-c2cccc(NS(=O)(=O)c3c(F)cccc3F)c2F)c(-c2ccnc(N)n2)s1 | Dabrafenib | 0.4625 | 0.7095 |

**Table S6** A list of antitumor SMIs and corresponding prediction results in GROVER-BBBp model. (continued)

| **SMILES** | **Drug** | **Predicted probability** | **Uncertainty** |
| --- | --- | --- | --- |
| COc1cc(OC)cc(N(CCNC(C)C)c2ccc3ncc(-c4cnn(C)c4)nc3c2)c1 | Erdafitinib | 0.3502 | 0.7113 |
| FC(F)(F)c1ccc(CNc2ccc(Cc3c[nH]c4ncc(Cl)cc34)cn2)cn1 | Pexidartinib | 0.6587 | 0.7117 |
| CN1CCC(COc2cnc(-c3cccc(Cn4nc(-c5cccc(C#N)c5)ccc4=O)c3)nc2)CC1 | Tepotinib | 0.6538 | 0.7134 |
| O=C(Nc1cnn2ccc(N3CCCC3c3cc(F)ccc3F)nc12)N1CCC(O)C1 | Larotrectinib | 0.6909 | 0.7149 |
| C#Cc1cccc(Nc2ncnc3cc(OCCOC)c(OCCOC)cc23)c1 | Erlotinib | 0.4307 | 0.7155 |
| Cn1cnc2c(F)c(Nc3ccc(Br)cc3F)c(C(=O)NOCCO)cc21 | Binimetinib | 0.6662 | 0.7197 |
| CCCS(=O)(=O)Nc1ccc(F)c(C(=O)c2c[nH]c3ncc(-c4ccc(Cl)cc4)cc23)c1F | Vemurafenib | 0.5782 | 0.7184 |
| CC(C)(O)CNc1nc(Nc2ccnc(C(F)(F)F)c2)nc(-c2cccc(C(F)(F)F)n2)n1 | Enasidenib | 0.4231 | 0.7194 |
| Cc1c(C(=O)Nc2ccc(N3CC(C)OC(C)C3)nc2)cccc1-c1ccc(OC(F)(F)F)cc1 | Sonidegib | 0.7141 | 0.7215 |
| CNC(=O)c1cc(Oc2ccc(NC(=O)Nc3ccc(Cl)c(C(F)(F)F)c3)c(F)c2)ccn1 | Regorafenib | 0.6339 | 0.7229 |
| Cn1cnc2c(F)c(Nc3ccc(Br)cc3Cl)c(C(=O)NOCCO)cc21 | Selumetinib | 0.6043 | 0.7233 |
| CC#CC(=O)N1CCCC1c1nc(-c2ccc(C(=O)Nc3ccccn3)cc2)c2c(N)nccn12 | Acalabrutinib | 0.6420 | 0.7241 |
| CCn1c(=O)c(-c2cc(NC(=O)Nc3ccccc3)c(F)cc2Br)cc2cnc(NC)cc21 | Ripretinib | 0.5477 | 0.7253 |
| COc1cc2nccc(Oc3ccc(NC(=O)NC4CC4)c(Cl)c3)c2cc1C(N)=O | Lenvatinib | 0.6462 | 0.7262 |
| CNC(=O)c1cc(Oc2ccc(NC(=O)Nc3ccc(Cl)c(C(F)(F)F)c3)cc2)ccn1 | Sorafenib | 0.6531 | 0.7271 |
| COc1cc2ncnc(Nc3ccc(F)c(Cl)c3)c2cc1NC(=O)C=CCN1CCCCC1 | Dacomitinib | 0.7081 | 0.7279 |
| CN1CCN(c2ccc(C(=O)Nc3n[nH]c4ccc(Cc5cc(F)cc(F)c5)cc34)c(NC3CCOCC3)c2)CC1 | Entrectinib | 0.6816 | 0.7286 |
| CC(Nc1ncnc2[nH]cnc12)c1cc2cccc(Cl)c2c(=O)n1-c1ccccc1 | Duvelisib | 0.4442 | 0.7282 |
| Cc1cn(-c2cc(NC(=O)c3ccc(C)c(Nc4nccc(-c5cccnc5)n4)c3)cc(C(F)(F)F)c2)cn1 | Nilotinib | 0.3436 | 0.7295 |

**Table S6** A list of antitumor SMIs and corresponding prediction results in GROVER-BBBp model. (continued)

| **SMILES** | **Drug** | **Predicted probability** | **Uncertainty** |
| --- | --- | --- | --- |
| CCN1CCN(Cc2ccc(Nc3ncc(F)c(-c4cc(F)c5nc(C)n(C(C)C)c5c4)n3)nc2)CC1 | Abemaciclib | 0.6547 | 0.7299 |
| Cc1nc(Nc2ncc(C(=O)Nc3c(C)cccc3Cl)s2)cc(N2CCN(CCO)CC2)n1 | Dasatinib | 0.3738 | 0.7342 |
| COc1cc(Nc2c(C#N)cnc3cc(OCCCN4CCN(C)CC4)c(OC)cc23)c(Cl)cc1Cl | Bosutinib | 0.6158 | 0.7372 |
| CN(C)C(=O)c1cc2cnc(Nc3ccc(N4CCNCC4)cn3)nc2n1C1CCCC1 | Ribociclib | 0.6399 | 0.7397 |
| CC(=O)c1c(C)c2cnc(Nc3ccc(N4CCNCC4)cn3)nc2n(C2CCCC2)c1=O | Palbociclib | 0.5571 | 0.7452 |
| CN(C)CC=CC(=O)Nc1cc2c(Nc3ccc(F)c(Cl)c3)ncnc2cc1OC1CCOC1 | Afatinib | 0.4016 | 0.7480 |
| C=CC(=O)Nc1cc(Nc2nccc(-c3cn(C)c4ccccc34)n2)c(OC)cc1N(C)CCN(C)C | Osimertinib | 0.4864 | 0.7486 |
| CC(C)(C)c1cc(NC(=O)Nc2ccc(-c3cn4c(n3)sc3cc(OCCN5CCOCC5)ccc34)cc2)no1 | Quizartinib | 0.6127 | 0.7488 |
| CCc1nc(C(N)=O)c(Nc2ccc(N3CCC(N4CCN(C)CC4)CC3)c(OC)c2)nc1NC1CCOCC1 | Gilteritinib | 0.4381 | 0.7512 |
| O=C(c1ccc(F)c(F)c1Nc1ccc(I)cc1F)N1CC(O)(C2CCCCN2)C1 | Cobimetinib | 0.5977 | 0.7556 |
| COc1cc(N2CCC(N3CCN(C)CC3)CC2)ccc1Nc1ncc(Cl)c(Nc2ccccc2P(C)(C)=O)n1 | Brigatinib | 0.3728 | 0.7557 |
| N#Cc1ccnc(N2C(=O)CCC2C(=O)N(c2cncc(F)c2)C(C(=O)NC2CC(F)(F)C2)c2ccccc2Cl)c1 | Ivosidenib | 0.6159 | 0.7604 |
| COc1ccc(CN2C3CC2CN(c2ccc(-c4cc(OCC(C)(C)O)cn5ncc(C#N)c45)cn2)C3)cn1 | Selpercatinib | 0.5280 | 0.7803 |

**Table S7** Prediction results for external dataset collected from Colclough [4] and Kim’s [5] works in GROVER-BBBp model.

| **SMILES** | **Name** | **BBB+/BBB-(K_p,uu_)** | **Predicted probability** | **Finetune predicted probability** |
| --- | --- | --- | --- | --- |
| CCN1CCN(Cc2ccc(-c3cc4c(NC(C)c5ccccc5)ncnc4[nH]3)cc2)CC1 | AEE788 | 0 | 0.7582 | 0.7418 |
| COc1cc2c(Nc3ccc(Br)cc3F)ncnc2cc1OCC1CCN(C)CC1 | Vandetanib | 1 | 0.8119 | 0.6994 |
| COc1cc2ncnc(Nc3cccc(Cl)c3F)c2cc1OC(=O)N1CCN(C)CC1C | AZD3759 | 1 | 0.4612 | 0.0970 |
| C#Cc1cccc(Nc2ncnc3cc(OCCOC)c(OCCOC)cc23)c1 | Erlotinib | 0 | 0.4307 | 0.0993 |
| C#Cc1cccc(Nc2ncnc3cc4c(cc23)OCCOCCOCCO4)c1 | Icotinib | 1 | 0.5472 | 0.5179 |
| C=CC(=O)N1CCC(Oc2cc3c(Nc4ccc(Cl)c(Cl)c4F)ncnc3cc2OC)CC1 | Poziotinib | 0 | 0.6923 | 0.1759 |
| C=CC(=O)N1CCC(Oc2nc(Nc3ccc(N4CCC(N5CCN(C)CC5)CC4)cc3)c(C(N)=O)nc2CC)C1 | Naquotinib | 0 | 0.5449 | 0.5307 |
| C=CC(=O)Nc1cc(Nc2nccc(-c3c[nH]c4ccccc34)n2)c(OC)cc1N(C)CCN(C)C | AZ’5104 | 0 | 0.4344 | 0.6978 |
| C=CC(=O)Nc1cc(Nc2nccc(-c3cn(C)c4ccccc34)n2)c(OC)cc1N(C)CCN(C)C | Osimertinib | 1 | 0.4864 | 0.6952 |
| C=CC(=O)Nc1cc(Nc2nccc(-n3cc(CN(C)C)c(-c4ccccc4)n3)n2)c(OC)cc1N1CCOCC1 | Lazertinib | 0 | 0.4290 | 0.7603 |
| C=CC(=O)Nc1cccc(Nc2nc(Nc3ccc(N4CCN(C(C)=O)CC4)cc3OC)ncc2C(F)(F)F)c1 | Rociletinib | 0 | 0.1660 | 0.4282 |
| C=CC(=O)Nc1cccc(Oc2nc(Nc3ccc(N4CCN(C)CC4)c(F)c3)nc3[nH]ccc23)c1 | Avitinib | 0 | 0.5345 | 0.1498 |
| C=CC(=O)Nc1cccc(Oc2nc(Nc3ccc(N4CCN(C)CC4)cc3)nc3ccsc23)c1 | Olmutinib | 0 | 0.7246 | 0.3955 |
| Cc1cc(C(=O)Nc2nc3cccc(Cl)c3n2C2CCCCN(C(=O)C=CCN(C)C)C2)ccn1 | Nazartinib | 0 | 0.8504 | 0.3976 |
| CN(C)CC=CC(=O)Nc1cc2c(Nc3ccc(F)c(Cl)c3)ncnc2cc1OC1CCOC1 | Afatinib | 0 | 0.4016 | 0.6171 |
| COc1cc2c(Nc3ccc(Cl)c(Cl)c3F)ncnc2cc1OCC1CC2CN(C)CC2C1 | Tesevatinib | 0 | 0.7136 | 0.6033 |
| COc1cc2ncnc(Nc3ccc(F)c(Cl)c3)c2cc1NC(=O)C=CCN1CCCCC1 | Dacomitinib | 0 | 0.7081 | 0.3957 |
| COc1cc2ncnc(Nc3ccc(F)c(Cl)c3)c2cc1OCCCN1CCOCC1 | Gefitinib | 0 | 0.7758 | 0.2882 |
|  |  |  | 7/18 | 11/18 |

**Table S8** The details of 9 drug-like datasets from admetSAR [6]

| **dataset** | **number of compounds** | **number of filtered compounds** | **+** | **-** |
| --- | --- | --- | --- | --- |
| fathead minnow toxicity | 554 | 553 | 366 | 187 |
| tetrahymena pyriformis toxicity | 1571 | 1324 | 1021 | 303 |
| AMES mutagenicity | 8445 | 6812 | 3138 | 3674 |
| hERG inhibitor (II) | 806 | 792 | 424 | 368 |
| CYP3A4 substrates | 674 | 666 | 354 | 312 |
| CYP1A2 inhibitor | 14903 | 14062 | 7211 | 6851 |
| Caco-2 permeability | 674 | 662 | 295 | 367 |
| P-gp inhibitor (I) | 1273 | 1272 | 792 | 475 |
| P-gp inhibitor (II) | 1275 | 1176 | 615 | 561 |

**Table S9** Model performance on fivefold cross-validations of 9 drug-like datasets in GROVER model.

| **dataset** | **ROC_AUC** | **PRC_AUC** | **MCC** | **BACC** |
| --- | --- | --- | --- | --- |
| fathead minnow toxicity | 0.8974 ± 0.0244 | 0.9405 ± 0.0138 | 0.6286 ± 0.0668 | 0.8100 ± 0.0317 |
| tetrahymena pyriformis toxicity | 0.9520 ± 0.0123 | 0.9847 ± 0.0046 | 0.7266 ± 0.0425 | 0.8516 ± 0.0316 |
| AMES mutagenicity | 0.8780 ± 0.0137 | 0.8916 ± 0.0140 | 0.6136 ± 0.0234 | 0.8059 ± 0.0114 |
| hERG inhibitor (II) | 0.8446 ± 0.0110 | 0.8604 ± 0.0354 | 0.5570 ± 0.0575 | 0.7777 ± 0.0300 |
| CYP3A4 substrates | 0.6706 ± 0.0257 | 0.6732 ± 0.0202 | 0.2788 ± 0.0500 | 0.6358 ± 0.0239 |
| CYP1A2 inhibitor | 0.8903 ± 0.0079 | 0.9005 ± 0.0087 | 0.6306 ± 0.0215 | 0.8153 ± 0.0108 |
| Caco-2 permeability | 0.8410 ± 0.0447 | 0.8241 ± 0.0581 | 0.5320 ± 0.1252 | 0.7630 ± 0.0600 |
| P-gp inhibitor (I) | 0.9030 ± 0.0219 | 0.9337 ± 0.0279 | 0.6352 ± 0.0394 | 0.8037 ± 0.0229 |
| P-gp inhibitor (II) | 0.9426 ± 0.0172 | 0.9538 ± 0.0185 | 0.7579 ± 0.0742 | 0.8782 ± 0.0373 |

**Table S10** Model performance on test set of 9 drug-like datasets in GROVER model.

| **dataset** | **ROC_AUC** | **PRC_AUC** | **MCC** | **BACC** |
| --- | --- | --- | --- | --- |
| fathead minnow toxicity | 0.9463 | 0.9709 | 0.6735 | 0.8457 |
| tetrahymena pyriformis toxicity | 0.9695 | 0.9910 | 0.7752 | 0.8853 |
| AMES mutagenicity | 0.8963 | 0.9063 | 0.6551 | 0.8270 |
| hERG inhibitor (II) | 0.9231 | 0.9318 | 0.6838 | 0.8423 |
| CYP3A4 substrates | 0.6798 | 0.6974 | 0.1680 | 0.5798 |
| CYP1A2 inhibitor | 0.8863 | 0.8997 | 0.6185 | 0.8093 |
| Caco-2 permeability | 0.9250 | 0.9250 | 0.6274 | 0.8092 |
| P-gp inhibitor (I) | 0.9243 | 0.9435 | 0.6965 | 0.8452 |
| P-gp inhibitor (II) | 0.9517 | 0.9524 | 0.8059 | 0.9029 |


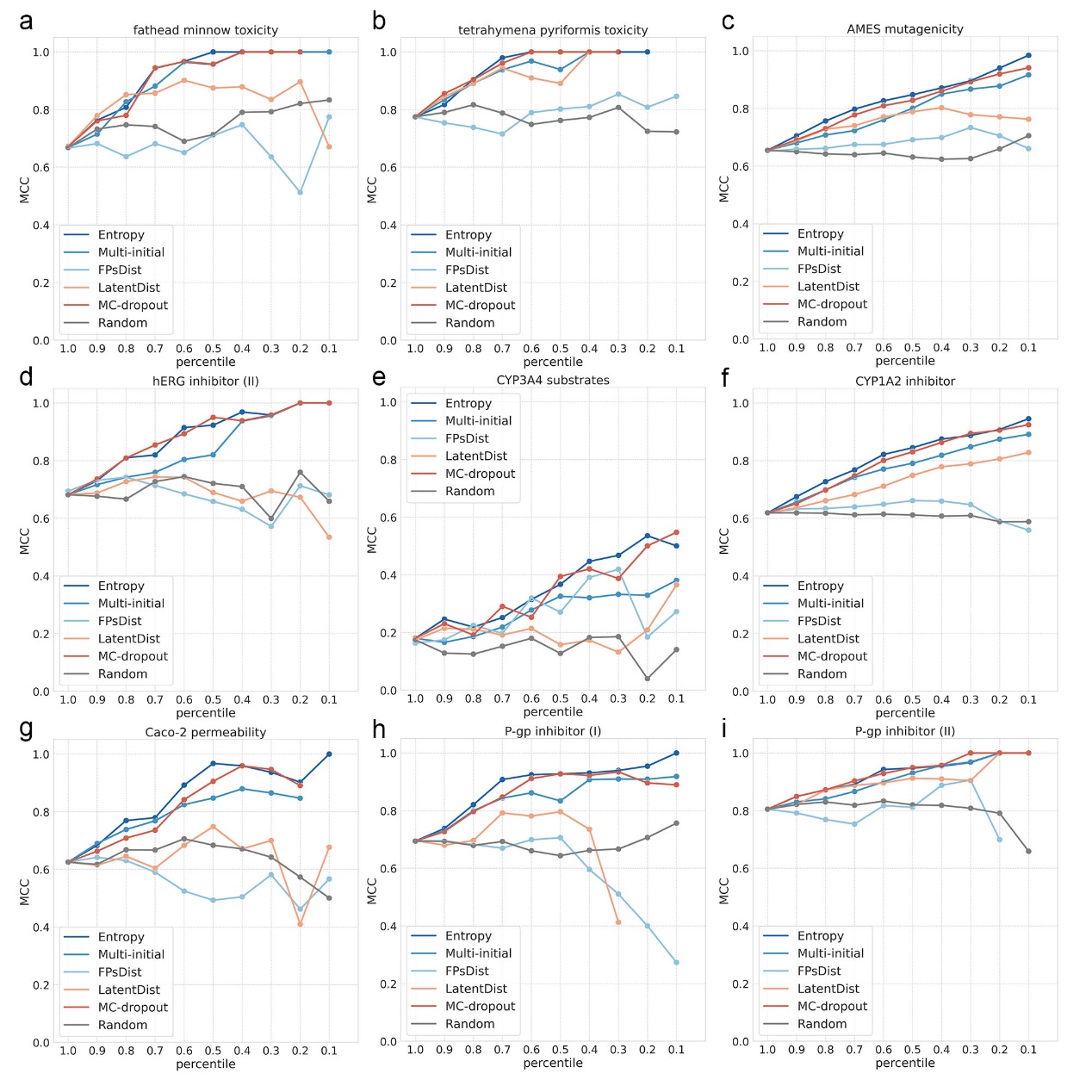


**Fig. S1** Prediction performance by introducing different uncertainty estimation methods in 9 GROVER models.

**Table S11** Model performance of various combination of uncertainty estimation methods on fathead minnow toxicity dataset in GROVER model.

| **Entropy** | **MC-dropout** | **Multi-initial** | **FPsDist** | **LatentDist** | **MCC_AUC** |
| --- | --- | --- | --- | --- | --- |
| √ |  |  |  |  | 0.8317 |
|  | √ |  |  |  | 0.8245 |
|  |  | √ |  |  | 0.8178 |
|  |  |  | √ |  | 0.5978 |
|  |  |  |  | √ | 0.7546 |
| √ | √ |  |  |  | **0.8339** |
| √ | √ | √ |  |  | 0.8333 |
| √ | √ |  | √ |  | 0.8333 |
| √ | √ |  |  | √ | 0.8330 |

**Table S12** Model performance of various combination of uncertainty estimation methods on tetrahymena pyriformis toxicity dataset in GROVER model.

| **Entropy** | **MC-dropout** | **Multi-initial** | **FPsDist** | **LatentDist** | **MCC_AUC** |
| --- | --- | --- | --- | --- | --- |
| √ |  |  |  |  | 0.8590 |
|  | √ |  |  |  | 0.8608 |
|  |  | √ |  |  | 0.8458 |
|  |  |  | √ |  | 0.7082 |
|  |  |  |  | √ | 0.8367 |
| √ | √ |  |  |  | **0.8641** |
| √ | √ | √ |  |  | 0.8613 |
| √ | √ |  | √ |  | 0.8641 |
| √ | √ |  |  | √ | 0.8638 |

**Table S13** Model performance of various combination of uncertainty estimation methods on AMES mutagenicity dataset in GROVER model.

| **Entropy** | **MC-dropout** | **Multi-initial** | **FPsDist** | **LatentDist** | **MCC_AUC** |
| --- | --- | --- | --- | --- | --- |
| √ |  |  |  |  | **0.7462** |
|  | √ |  |  |  | 0.7308 |
|  |  | √ |  |  | 0.7054 |
|  |  |  | √ |  | 0.6158 |
|  |  |  |  | √ | 0.6777 |
| √ | √ |  |  |  | 0.7447 |
| √ | √ | √ |  |  | 0.7400 |
| √ | √ |  | √ |  | 0.7458 |
| √ | √ |  |  | √ | 0.7420 |

**Table S14** Model performance of various combination of uncertainty estimation methods on hERG inhibitor (II) dataset in GROVER model.

| **Entropy** | **MC-dropout** | **Multi-initial** | **FPsDist** | **LatentDist** | **MCC_AUC** |
| --- | --- | --- | --- | --- | --- |
| √ |  |  |  |  | 0.7966 |
|  | √ |  |  |  | **0.7981** |
|  |  | √ |  |  | 0.7576 |
|  |  |  | √ |  | 0.6136 |
|  |  |  |  | √ | 0.6226 |
| √ | √ |  |  |  | 0.7937 |
| √ | √ | √ |  |  | 0.7916 |
| √ | √ |  | √ |  | 0.7951 |
| √ | √ |  |  | √ | 0.7977 |

**Table S15** Model performance of various combination of uncertainty estimation methods on CYP3A4 substrates dataset in GROVER model.

| **Entropy** | **MC-dropout** | **Multi-initial** | **FPsDist** | **LatentDist** | **MCC_AUC** |
| --- | --- | --- | --- | --- | --- |
| √ |  |  |  |  | 0.3191 |
|  | √ |  |  |  | 0.3033 |
|  |  | √ |  |  | 0.2439 |
|  |  |  | √ |  | 0.2401 |
|  |  |  |  | √ | 0.1774 |
| √ | √ |  |  |  | 0.3263 |
| √ | √ | √ |  |  | 0.3162 |
| √ | √ |  | √ |  | **0.3439** |
| √ | √ |  |  | √ | 0.2570 |

**Table S16** Model performance of various combination of uncertainty estimation methods on CYP1A2 inhibitor dataset in GROVER model.

| **Entropy** | **MC-dropout** | **Multi-initial** | **FPsDist** | **LatentDist** | **MCC_AUC** |
| --- | --- | --- | --- | --- | --- |
| √ |  |  |  |  | **0.7289** |
|  | √ |  |  |  | 0.7163 |
|  |  | √ |  |  | 0.6955 |
|  |  |  | √ |  | 0.5704 |
|  |  |  |  | √ | 0.6539 |
| √ | √ |  |  |  | 0.7254 |
| √ | √ | √ |  |  | 0.7245 |
| √ | √ |  | √ |  | 0.7179 |
| √ | √ |  |  | √ | 0.7212 |

**Table S17** Model performance of various combination of uncertainty estimation methods on Caco-2 permeability dataset in GROVER model.

| **Entropy** | **MC-dropout** | **Multi-initial** | **FPsDist** | **LatentDist** | **MCC_AUC** |
| --- | --- | --- | --- | --- | --- |
| √ |  |  |  |  | 0.7703 |
|  | √ |  |  |  | 0.7464 |
|  |  | √ |  |  | 0.7273 |
|  |  |  | √ |  | 0.5025 |
|  |  |  |  | √ | 0.5728 |
| √ | √ |  |  |  | 0.7581 |
| √ |  | √ |  |  | **0.7802** |
| √ | √ | √ |  |  | 0.7648 |
| √ | √ |  | √ |  | 0.7550 |
| √ | √ |  |  | √ | 0.7483 |

**Table S18** Model performance of various combination of uncertainty estimation methods on P-gp inhibitor (I) dataset in GROVER model.

| **Entropy** | **MC-dropout** | **Multi-initial** | **FPsDist** | **LatentDist** | **MCC_AUC** |
| --- | --- | --- | --- | --- | --- |
| √ |  |  |  |  | **0.7990** |
|  | √ |  |  |  | 0.7755 |
|  |  | √ |  |  | 0.7602 |
|  |  |  | √ |  | 0.5444 |
|  |  |  |  | √ | 0.6742 |
| √ | √ |  |  |  | 0.7859 |
| √ | √ | √ |  |  | 0.7883 |
| √ | √ |  | √ |  | 0.7880 |
| √ | √ |  |  | √ | 0.7834 |

**Table S19** Model performance of various combination of uncertainty estimation methods on P-gp inhibitor (II) dataset in GROVER model.

| **Entropy** | **MC-dropout** | **Multi-initial** | **FPsDist** | **LatentDist** | **MCC_AUC** |
| --- | --- | --- | --- | --- | --- |
| √ |  |  |  |  | 0.8326 |
|  | √ |  |  |  | 0.8362 |
|  |  | √ |  |  | 0.8196 |
|  |  |  | √ |  | 0.7340 |
|  |  |  |  | √ | 0.8105 |
| √ | √ |  |  |  | 0.8342 |
| √ | √ | √ |  |  | 0.8310 |
| √ | √ |  | √ |  | **0.8368** |
| √ | √ |  |  | √ | 0.8351 |

**References**

1. Shaikh N, Sharma M, Garg P (2017) Selective Fusion of Heterogeneous Classifiers for Predicting Substrates of Membrane Transporters. J Chem Inf Model 57:594-607.

2. Wang X, Zhu X, Ye M, Wang Y, Li CD, Xiong Y, Wei DQ (2019) STS-NLSP: A Network-Based Label Space Partition Method for Predicting the Specificity of Membrane Transporter Substrates Using A Hybrid Feature of Structural and Semantic Similarity. Front Bioeng Biotechnol 7:306.

3. Schyman P, Liu R, Desai V, Wallqvist A (2017) vNN Web Server for ADMET Predictions. Front Pharmacol 8:889.

4. Colclough N, Chen K, Johnstrom P, Strittmatter N, Yan Y, Wrigley GL, Schou M, Goodwin R, Varnas K, Adua SJ *et al* (2021) Preclinical Comparison of the Blood-Brain Barrier Permeability of Osimertinib with Other EGFR TKIs. Clin Cancer Res 27:189-201.

5. Kim M, Laramy JK, Mohammad AS, Talele S, Fisher J, Sarkaria JN, Elmquist WF (2019) Brain Distribution of A Panel of Epidermal Growth Factor Receptor Inhibitors Using Cassette Dosing in Wild-Type and Abcb1/Abcg2-Deficient Mice. Drug Metab Dispos 47:393-404.

6. Cheng F, Li W, Zhou Y, Shen J, Wu Z, Liu G, Lee PW, Tang Y (2012) admetSAR: a comprehensive source and free tool for assessment of chemical ADMET properties. J Chem Inf Model 52:3099-3105.
